# Inhibition of cardiac PERK signaling promotes peripartum cardiac dysfunction

**DOI:** 10.1038/s41598-021-98344-7

**Published:** 2021-09-21

**Authors:** Takashi Shimizu, Akashi Taguchi, Yoshiki Higashijima, Yasuharu Kanki, Ryo Nakaki, Yoshihiro Urade, Youichiro Wada

**Affiliations:** 1 Isotope Science Center, The University of Tokyo, Tokyo, 113-0032 Japan; 2 Department of Cardiovascular Medicine, The University of Tokyo Graduate School of Medicine, Tokyo, 113-8655 Japan; 3 Department of Pathology, Brigham and Women’s Hospital, Harvard Medical School, Boston, MA 02115 USA; 4 Department of Bioinformational Pharmacology, Tokyo Medical and Dental University, Tokyo, 113-8510 Japan; 5 Department of Proteomics, Faculty of Health and Medical Sciences, The Novo Nordisk Foundation Center for Protein Research, University of Copenhagen, Blegdamsvej 3B, 2200 Copenhagen, Denmark; 6 Laboratory of Laboratory/Sports Medicine, Division of Clinical Medicine, Faculty of Medicine, University of Tsukuba, 1-1-1 Tennodai, Tsukuba, Ibaraki 305-8577 Japan; 7 Rhelixa Inc., Tokyo, 101-0061 Japan; 8 Center for Supporting Pharmaceutical Education, Daiichi University of Pharmacy, 22-1 Tamagawa-machi, Minami-ku, Fukuoka, 815-8511 Japan

**Keywords:** Cardiology, Cardiovascular biology, Cardiovascular diseases, Heart failure

## Abstract

Peripartum cardiomyopathy (PPCM) is a life-threatening heart failure occurring in the peripartum period. Although mal-angiogenesis, induced by the 16-kDa N-terminal prolactin fragment (16 K PRL), is involved in the pathogenesis, the effect of full-length prolactin (23 K PRL) is poorly understood. We transfected neonate rat cardiomyocytes with plasmids containing 23 K PRL or 16 K PRL in vitro and found that 23 K PRL, but not 16 K PRL, upregulated protein kinase RNA-like endoplasmic reticulum kinase (PERK) signaling, and hypoxia promoted this effect. During the perinatal period, cardiomyocyte-specific PERK homogenous knockout (CM-KO) mice showed PPCM phenotypes after consecutive deliveries. Downregulation of PERK or JAK/STAT signaling and upregulation of apoptosis were observed in CM-KO mouse hearts. Moreover, in bromocriptine-treated CM-KO mice, cardiac function did not improve and cardiomyocyte apoptosis was not suppressed during the peripartum period. These results demonstrate that interaction between 23 K PRL and PERK signaling is cardioprotective during the peripartum term.

## Introduction

Peripartum cardiomyopathy (PPCM) is a potentially life-threatening condition that typically presents as heart failure (HF) with reduced ejection fraction in the last month of pregnancy or in the months following delivery in women without other known causes of HF^1^. It is a diagnosis of exclusion, and the etiology remains unknown. In the postpartum maternal heart, deregulation of multiple upstream factors, such as STAT3^2^ or AKT^3^, accelerates massive cardiac hypertrophy and diminishes capillary density, metabolic remodeling, and cardiac inflammation. In this condition, physiologically rising oxidative stress leads to the enhanced generation of reactive oxygen species (ROS). The enhanced oxidative stress promotes activation of proteases, such as cathepsin D and metalloproteinases (MMPSs), which subsequently cleave the full-length nursing hormone prolactin (23 K PRL) into the antiangiogenic N-terminal PRL metabolite (16 K PRL). In turn, 16 K PRL induces the expression of nuclear factor kappa light-chain-enhancer of activated B cells (NFκB) and microRNA-146a (miR-146a) in endothelial cells, resulting in the downregulation of Erb-B2 receptor tyrosine kinase 4 (ERBB4) in cardiomyocytes^4^. Additionally, it is reported that 23 K PRL protects cardiomyocytes undergoing hypoxia through STAT3 signaling^5^; however, the molecular mechanism remains unclear. ROS overproduction also causes the misfolding of proteins in the endoplasmic reticulum (ER), leading to ER stress^6^. Three ER stress sensors, namely inositol-requiring enzyme 1 α (IRE1α), activating transcription factor-6 (ATF6), and protein kinase RNA (PKR)-like ER kinase (PERK), then initiate the unfolded protein response (UPR). We recently reported that chronic HF suppresses the PERK branch of UPR, but not the other branches, whereas acute HF promoted all branches^7^. PERK phosphorylates the α subunit of eukaryotic initiation factor 2 (eIF2α), leading to the activation of EIF2 signaling, which is related to protein translation and apoptosis^8^. The translation of *STAT3* is regulated by phosphorylated (p)-eIF2α^9^, and the phosphorylation of STAT3 is induced by PERK activation^10^. Taken together, PERK contributes to 23 K PRL-mediated cardioprotection along with STAT3. This study investigated the potential roles of the PRL-PERK axis in peripartum hearts.

## Results

### PERK signaling is upregulated by 23 K PRL, but not 16 K PRL, in neonatal rat cardiomyocytes (NRCMs)

**Figure 1:**
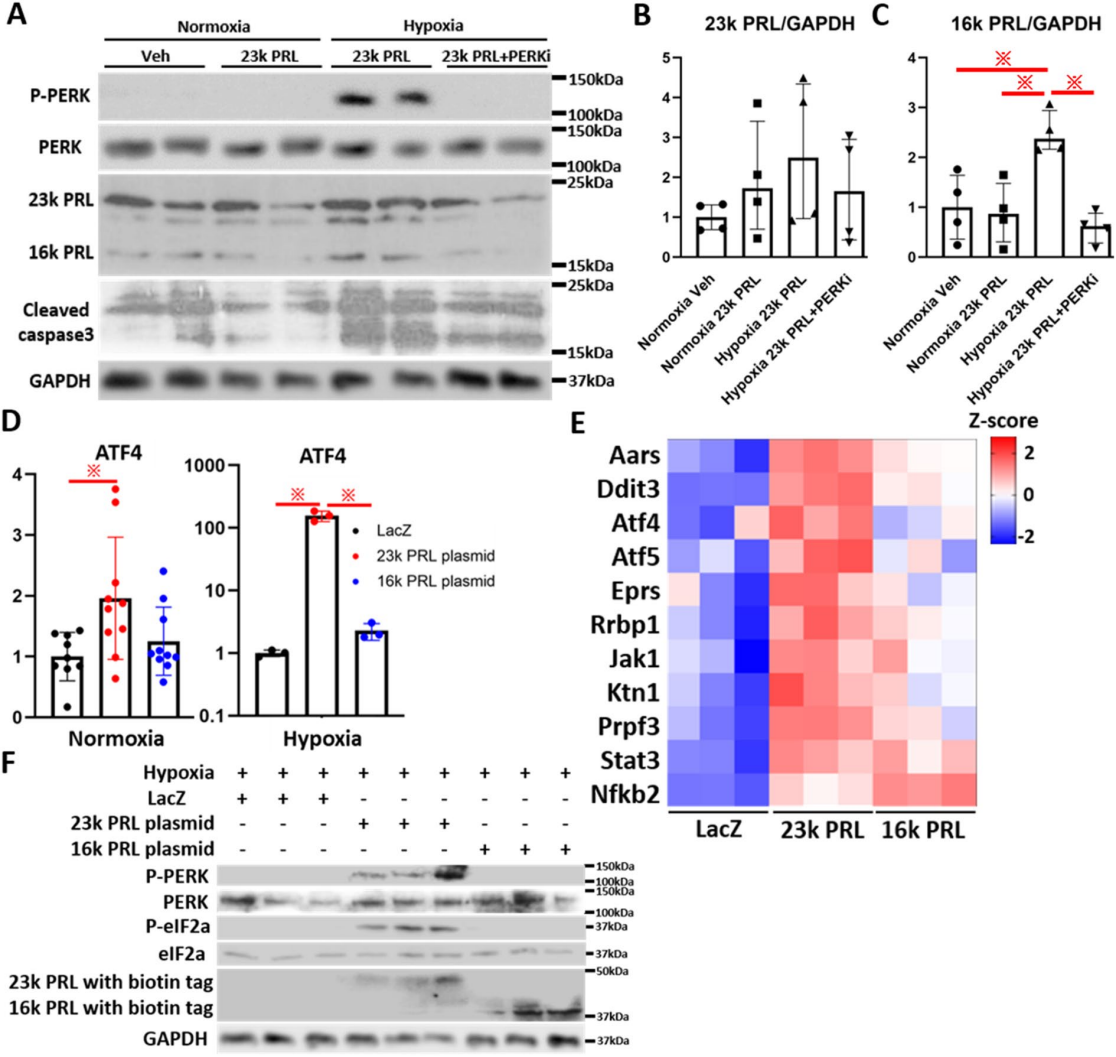
Full-length prolactin (23 k PRL) and hypoxia activates PERK signaling. (**A**) Protein levels of p-PERK, PERK, PRL (23 k and 16 k PRL), cleaved caspase3 and GAPDH in NRCMs under normoxia or hypoxia (O2 4% 24 h) treated with or without rat recombinant 23 k PRL (50 nM) or PERK inhibitor (PERKi, 1 μM) for 24 h. (**B**,**C**) Densitometric quantification of the 23 k PRL (**B**) or 16 k PRL (**C**) to GAPDH protein ratio (n = 4 cells per group). Data represent mean ± SD; *P* values were measured by one-way ANOVA with Bonferroni correction. (**D**) mRNA levels of ATF4 in normoxic or hypoxic NRCMs transfected with LacZ, 23 k or 16 k PRL coding plasmids. n = 3–10 sets of cells per group. Data represent mean ± SD.; **※** P < 0.05 one-way ANOVA with Bonferroni correction. (**E**) Representative PERK target genes in NRCMs with each plasmid under normoxia. n = 3 sets of cells per group. All genes were siginificantly (*P* < 0.05) upregulated in those with 23 k or 16 k PRL plasmid, compared with those with LacZ. Statistical tests used were one-way ANOVA with Bonferroni correction. (**F**) Using NRCMs with each plasmid under under hypoxia, protein levels of p-PERK, PERK, p-eIF2a, eIF2a, PRL (23 k and 16 k PRL) and GAPDH were shown. n = 3 sets of cells per group.

To investigate the potential roles of the 23 K PRL-PERK axis in PPCM, we treated NRCMs with recombinant 23 K PRL (50 nM) under normoxia or hypoxia (4% O_2_, 24 h) (Fig. 1A). The phosphorylation of PERK occurred to a greater extent under hypoxia, but not under normoxia. The cleavage of 23 K PRL into 16 K PRL was also induced under hypoxia (Fig. 1A–C). These effects were reversed by treatment with a PERK inhibitor (PERKi, 1 μM). The expression of cleaved caspase 3, which is related to apoptosis, was increased in hypoxic NRCMs with 23 K PRL treatment compared to normoxic cells with or without the treatment. PERKi treatment did not affect the expression of 23 k PRL in hypoxic NRCMs.

Next, to discriminate the effect of 23 K PRL from that of 16 K PRL, we transfected NRCMs with plasmids containing *LacZ*, 23 K PRL, or 16 K PRL. The expression of *Atf4*, one of PERK target genes, was increased slightly in 23 K PRL-expressing cells under normoxia compared to LacZ-expressing cells (Fig. 1D). Moreover, *Atf4* expression was strongly upregulated in 23 K PRL-expressing cells under hypoxia. RNA sequencing (RNA-seq) was performed to confirm the effect of PERK activation in these cells. All RNA-Seq results are provided in Table S1. A total of 12,371 genes exhibited more than fivefold increase in expression in at least one of the replicates in all the groups. We selected 11 PERK target genes, as described^9^ (Fig. 1E). The expression of *Aars, Ddit3, Atf4, Atf5, Eprs,* and *Rrbp1* was the highest among all mRNAs in 23 K PRL-expressing cells whereas that of *Jak1, Ktn1, Prpf3,* and *Stat3* was increased in 23 K or 16 K PRL-expressing cells compared to LacZ-expressing cells. The expression of *Nfkb2* was the highest in 16 K PRL-expressing cells.

Under hypoxic conditions, the phosphorylation of PERK and eIF2α was promoted by 23 K PRL-containing plasmid but not by 16 K PRL-containing plasmid (Fig. 1F). Taken together, these findings indicate that 23 K PRL activated PERK signaling, especially under hypoxia.

### PPCM-like phenotype in postpartum *PERK* knockout mice

**Figure 2:**
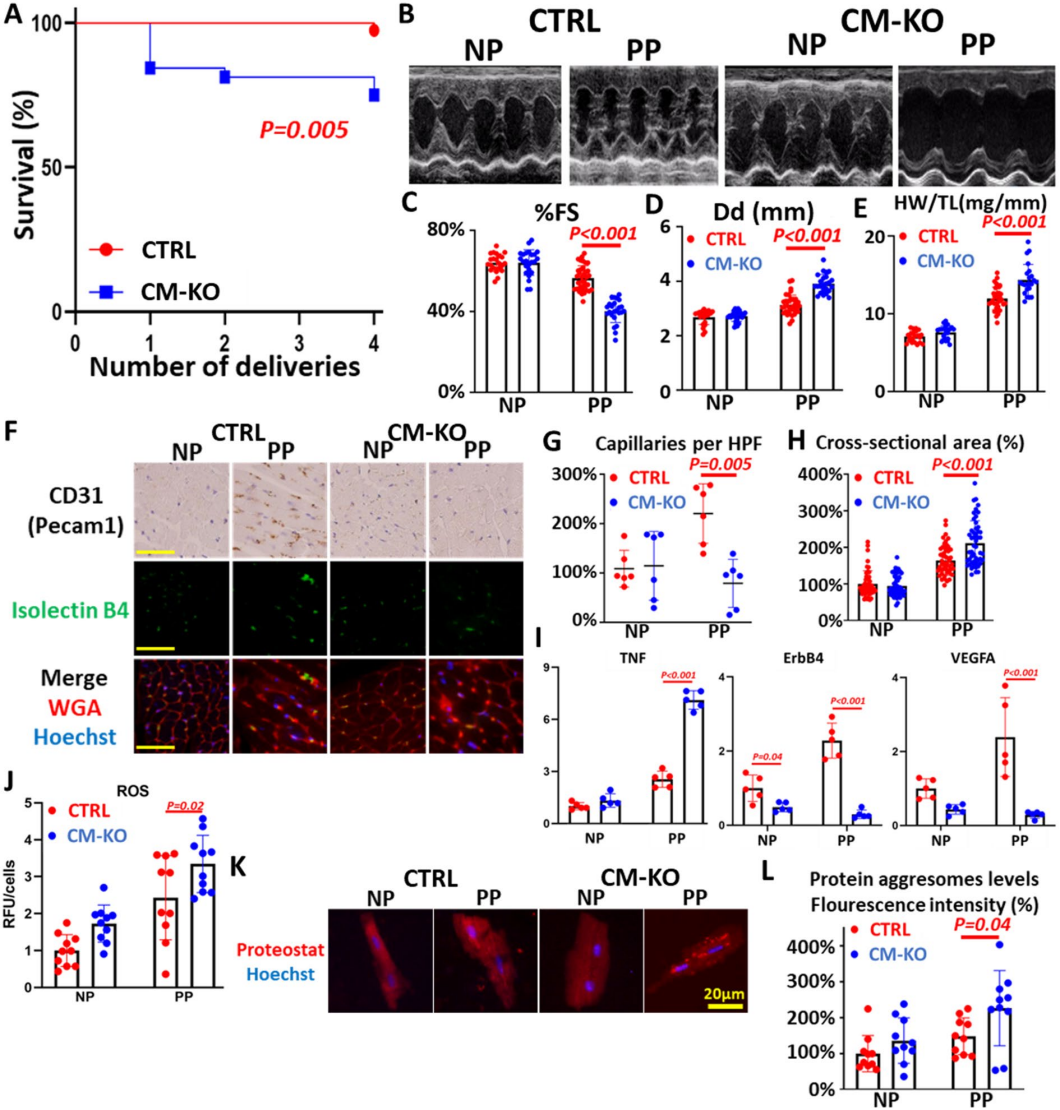
Cardiac-specific PERK homogenous knockout (CM-KO) mice develop peri-partum cardiomyopathy (PPCM). (**A**) Kaplan–Meier survival curve in CM-KO (n = 32) female mice versus control (CTRL, PERK lox/lox, n = 39) female mice. Log-rank test was performed between them. (**B**) Representative echocardiographic images in mice of each group. (**C**) (D) %FS (**C**) and Left ventricular end-diastolic dimension (Dd, **D**) in mice of each group; nulliparous (NP) CTRL (n = 22 mice), postpartum (PP) CTRL (n = 37 mice), NP CM-KO (n = 24 mice), and PP CM-KO (n = 24 mice). (**E**) Heart weight:tibial length (HW/TL) ratios of mice in each group. (**F**) CD31 staining of hearts in each group. Cardiac sections stained with isolectin B4 (blood vessels, green), WGA (cell membranes, red), and nuclei (Hoechst, blue). Scale bars: 50 μm. (**G**) Capillary density was quantified in HPF (high power field). n = 6 mice per group. (**H**) Quantification of cardiomyocyte cross-sectional area. n = 50 sets of cells per group. (**I**) The expressions of TNF, ErbB4 and Vegfa in hearts of each group; all normalized to GAPDH (n = 5 mice per group). Data represent mean ± SD.; *P* values were measured by two-way ANOVA with Bonferroni correction. (**J**) The levels of ROS in isolated cardiomyocytes were assessed by ELISA (n = 10 sets of cells per group). (**K**) Using the aggresome-specific ProteoStat dye, which showed an enhanced fluorescence signal of aggregated protein accumulation. Scale bars: 20 μm. (**L**) Quantification of fluorescence intensities in isolated cardiomyocytes from heats in each group. n = 10 sets of cells per group.

We generated and analyzed cardiomyocyte-specific *PERK* knockout (CM-KO) mice. Although female CM-KO mice were indistinguishable from the control (CTRL, *PERK* flox) mice at baseline (blood pressure, body weight, body temperature, and pupils per litter shown in Fig. S1A–D), these mice developed HF during the peripartum period. They died after multiple pregnancies (Fig. 2A). Echocardiographic studies revealed severely impaired cardiac function (percentage of fractional shortening, %FS) and markedly dilatated heart (Dd) in CM-KO mice compared to CTRL mice after their fourth delivery (PP) (Fig. 2B–D). Heart weight (HW/TL: heart weight normalized to tibia length) of CM-KO mice was comparable to that of CTRL at baseline (NP) but was significantly increased after fourth delivery (Fig. 2E). Furthermore, the capillary density was measured by staining with the endothelial-specific marker CD31 (Pecam1) and isolectin B4 (Fig. 2F,G). CM-KO PP hearts presented with an impaired cardiac angiogenic response compared to CTRL PP. The expression of *Vegfa* in CM-KO PP hearts was suppressed, compared with CTRL PP (Fig. 2H).

Quantification of the cardiomyocyte cross-sectional area after staining the cardiomyocyte membrane with tetramethyl rhodamine isothiocyanate-conjugated wheat-germ agglutinin (WGA) showed that CM-KO PP cardiomyocytes were enlarged compared to CTRL PP (Fig. 2F,I). These results suggest that PERK is critically involved in the regulation of cardiac angiogenic and hypertrophic responses.

### Cardiomyocyte-specific *PERK* deletion leads to ROS and protein aggresome accumulation

We investigated the ROS levels in isolated cardiomyocytes from these mice. The ROS levels were more upregulated in CM-KO PP mice compared to CTRL PP mice (Fig. 2J). Next, we evaluated unfolded protein accumulation in cardiomyocytes isolated from the hearts of mice, using the newly available aggresome-specific dye ProteoStat, which enhances the fluorescence signal of aggregated protein accumulation. An increased amount of unfolded proteins were accumulated in cells from CM-KO PP mice compared to CT PP mice (Fig. 2K,L).

### Genes involved in pathways of translation and oxidative stress were enhanced during the postpartum term

RNA-Seq was performed to investigate changes in the transcriptome during the postpartum term in CTRL and CM-KO mice. Each analysis was performed with whole myocardial tissues obtained at the study endpoint (after four consecutive deliveries). All RNA-Seq results are provided in Table S2. A total of 8865 genes exhibited more than fivefold increase in expression in at least one of the replicates in all groups (CTRL NP, CTRL PP, CM-KO NP, and CM-KO PP).

**Figure 3:**
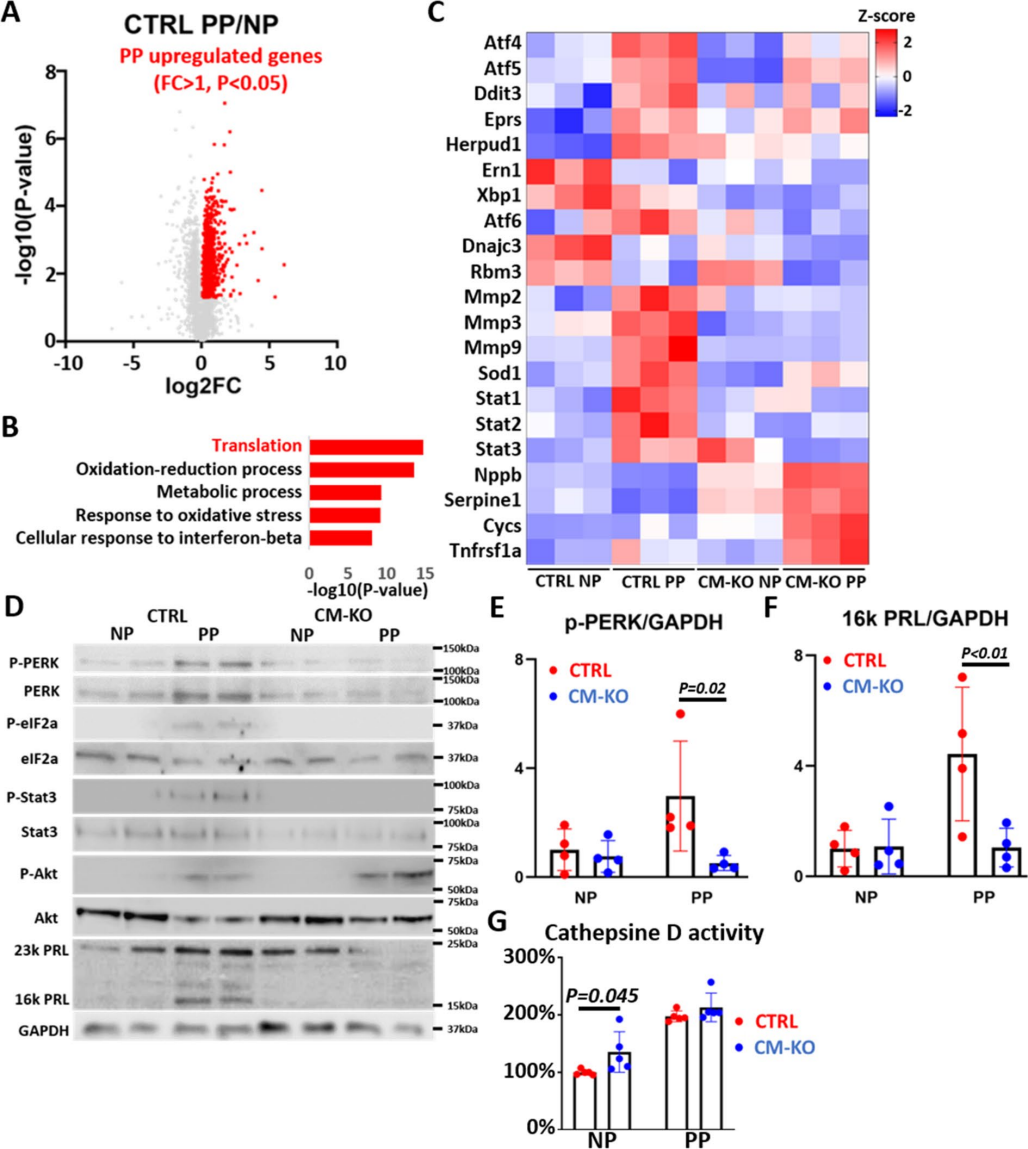
Comparison of hearts after consecutive deliveries with or without PERK deletion, using RNA sequencing. LV myocardium from NP or PP CTRL and CM-KO mice were subjected to RNA sequencing and subsequent differential expression analysis (n = 3 mice per group). (**A**) Volcano plot of RNA sequencing data, depicting mRNA data *P* values, calculated using the unpaired t test, versus fold change (FC) in comparisons between CTRL NP and CTRL PP mice. Upregulated (FC > 1) and different (*P* < 0.05) geneswere defined as “PP upregulated genes (red)”. (**B**) DAVID functional Gene Ontology analysis of biological processes for PP upregulated genes. Top5 enrichments were shown. (**C**) Representative genes related to (**B**) enrichments, were depicted. Statistical tests used were one-way ANOVA with Bonferroni correction. (**D**) Western blot analysis of phosphorylated PERK (p-PERK), PERK, phosphorylated eIF2a (p-eIF2a), eIF2a, phosphorylated Stat3 (p-Stat3), Stat3, phosphorylated Akt (p-Akt), Akt, and prolactin (23 k PRL, 16 k PRL) in hearts of each group. n = 4 mice per group. (**E,F**) Densitometric quantification of the p-PERK (**E**) or 16 k PRL (**F**) to GAPDH protein ratio (n = 4 mice per group). Data represent mean ± SD.; *P* values were measured by two-way ANOVA with Bonferroni correction. (**G**) Cathepsin D activity in heats. n = 10 mice per groups. Data represent mean ± SD; *P* values were measured by two-way ANOVA with Bonferroni correction.

First, we compared gene expression between CTRL NP and CTRL PP (Fig. 3A). Upregulated genes (FC > 1 and *P* < 0.05) were defined as “PP upregulated genes” (Fig. 3A).

Gene enrichment and functional annotation analyses were performed on these genes, using the DAVID tool (Fig. 3B). In Gene Ontology analysis, genes involved in translation, oxidation–reduction process, metabolic process, response to oxidative stress, and cellular response to interferon-beta were the most enriched.

### *PERK* deletion strongly modulates gene expression in PP mice

To investigate the relationship between the transcriptomic analysis and pathway analysis, we depicted representative genes on heatmaps (Fig. 3C). The expression of *Dnajc3*^11^ and *Rbm3*^12^ (PERK inhibitors) was inhibited in CTRL or CM-KO PP compared to CTRL NP, resulting in enhanced PERK signaling (related to translation) in the peripartum period. The PERK target genes^9^, Atf4, Atf5, Ddit3, Eprs, Herpud1, and Stat39, found to be upregulated in PP groups. The expression of *Atf4, Herpud1,* and *Stat3* was decreased in CM-KO PP compared to CTRL PP; however, the expression of other genes did not differ between them*. Ern1, Xbp1*, and *Atf6* belonged to the other UPR branches. The expression of *Ern1* was the highest in CTRL NP. The expression of *Xbp1* was suppressed in CM-KO NP or PP compared to CTRL NP or PP, respectively. The expression of *Atf6* was inhibited in CM-KO PP compared to CTRL PP. The expression of PP upregulated genes such as *Mmp2, Mmp3, Mmp9, Sod1, Stat1,* and *Stat2* was the highest in CTRL PP. *Mmp2, Mmp3, Mmp9,* and *Sod1* were involved in the oxidation–reduction process and response to oxidative stress. *Stat1* and *Stat2* were involved in the cellular response to interferon-beta. The expression of *Nppb, Serpine1, Cycs,* and *Tnfrsf1a* was the highest in CM-KO PP. *Nppb* is a biomarker for HF. PAI-1 (*Serpine1*) is required as a co-factor for 16 K PRL signaling^13^. CYCS belongs to the cytochrome c family of proteins and plays a major role in cell apoptosis. TNFRSF1A binds to TNF, leading to apoptosis. The expression of TNF was increased in CM-KO PP mice compared to CTRL PP mice (Fig. 2I). Overall, the expression of genes related to translation, oxidative stress, and inflammation was decreased in CM-KO PP mice, while those related to HF and apoptosis were upregulated.

### Cleavage of 23 K PRL was not observed in the hearts or sera of CM-KO PP mice

To further study PERK signaling activity, we performed western blotting analysis and observed increased phosphorylation of PERK, eIF2a, STAT3, and AKT in heart tissues of CTRL PP mice compared to CTRL NP mice (Fig. 3D). However, the levels of p-PERK were suppressed in CM-KO PP mice compared to CTRL PP mice (Fig. 3E). Previous studies have suggested that many enzymes, including cathepsin D^2^ and MMPSs^14^, lead to cleavage of 23 K PRL into 16 K PRL. We found an upregulation of 16 K PRL expression in the hearts (Fig. 3D,F) and sera (Fig. S1E) of PP CTRL mice, but not in CM-KO PP mice, whereas cathepsin D activity was not different between the hearts of CTRL PP mice and CM-KO PP mice (Fig. 3G). However, the expression of MMP-2, -3, and -9 in CTRL PP mice was the most upregulated among all groups (Fig. 3C). Thus, MMPS-mediated cleavage of 23 K PRL into 16 K PRL was suppressed in CM-KO PP mice. It was shown that 16 K PRL signaling inhibited the expression of ERBB4^4^, whereas STAT3 promoted it^15^. Using qRT-PCR, we confirmed that the expression of ERBB4 was suppressed in CM-KO mice compared to CTRL mice under both NP and PP conditions (Fig. 2I). As a result, 16 K PRL signaling was not advanced in CM-KO PP mice.

### Bromocriptine (BCR) does not ameliorate cardiac function during l the postpartum term in CM-KO mice

**Figure 4:**
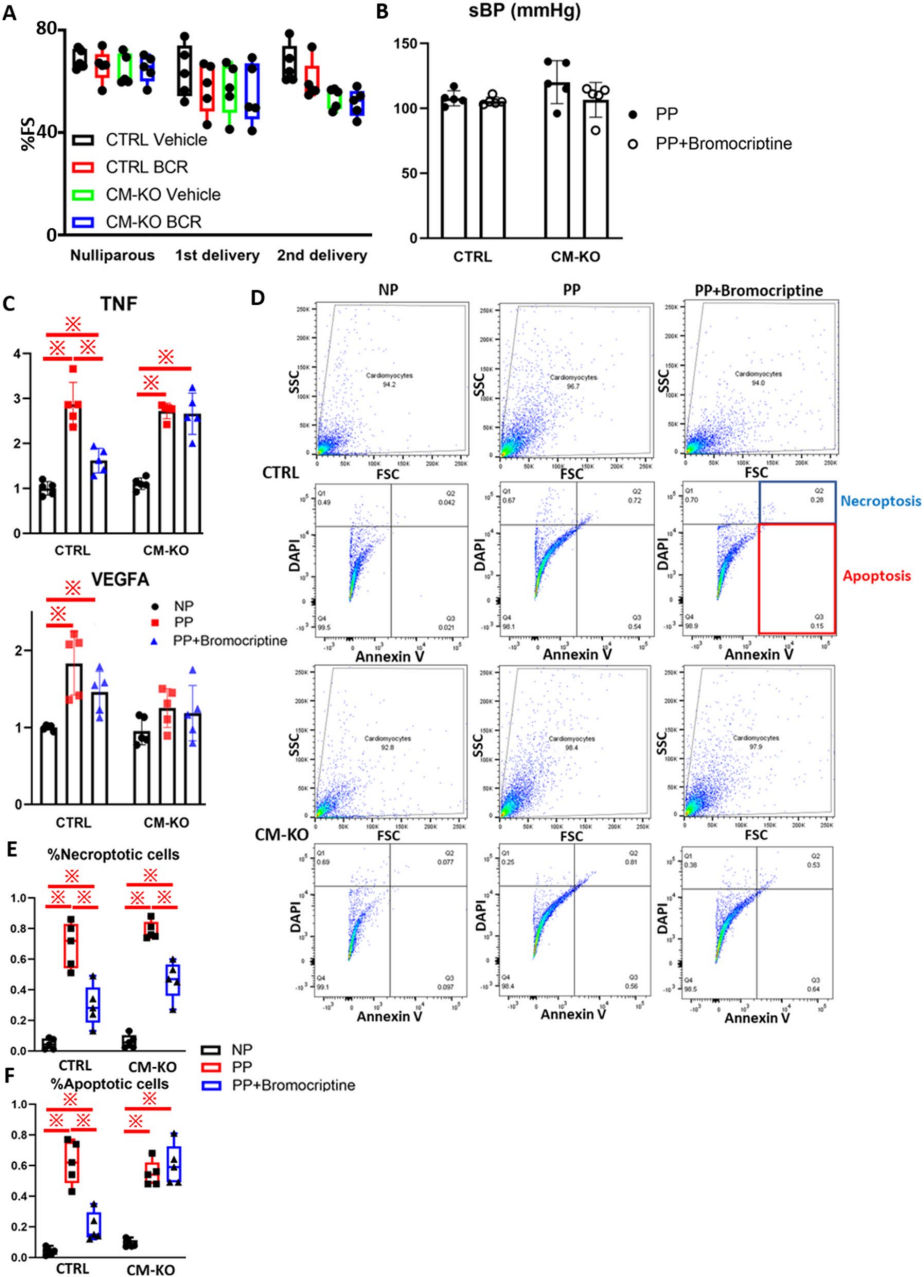
Bromocriptine (BCR) suppresses apoptosis in CTRL PP mice, but not CM-KO PP mice during. (**A**) Serial changes in %FS in CTRL and CM-KO mice with vehicle or bromocriptine (BCR) during the second delivery (n = 5 mice in each group). (**B,C**) systolic blood pressure (sBP, **B**) and the cardiac TNF and VEGFA levels (**C**) in CTRL and CM-KO mice after second delivery (n = 5 mice in each group). (**D**) Representative flow cytometric images in isolated cardiomyocytes of each mouse group (n = 5 mice in each group). (**E,F**) Quantification of necroptotic (DAPI^+^/Annexin V^+^, E), apoptotic (DAPI^+^/Annexin V^+^, F) cardiomyocytes of each mouse group (n = 5 mice in each group). Data represent mean ± SD; ※*P* < 0.05 two-way ANOVA with Bonferroni correction.

Previous studies have shown that anti-PRL agents, such as BCR, a dopamine D2 receptor agonist, not only improved cardiac function by reducing the plasma 16 K PRL levels^2^ but also suppressed cardiac hypertrophy by suppressing the plasma aldosterone and 23 K PRL levels during the lactation term^16^. We examined the effect of BCR in two consecutive deliveries in CTRL or CM-KO mice. BCR treatment did not affect cardiac function in these mice during the first or second postpartum term (Fig. 4A). BCR treatment did not change systolic blood pressure (sBP) in any group (Fig. 4B). We then examined the cardiac TNF levels using qRT-PCR. BCR treatment was found to inhibit TNF expression in CTRL PP mice but not in CM-KO PP mice (Fig. 4C). BCR treatment did not affect the expression of VEGFA neither in CTRL nor CM-KO PP mice. Next, we isolated cardiomyocytes from these mice after the second delivery to examine the presence of necroptotic or apoptotic cells. We measured the populations of necroptotic (DAPI^+^/Annexin V^+^) or apoptotic (DAPI^−^/Annexin V^+^) cardiomyocytes by flow cytometry (Fig. 4D). The populations of necroptotic cells were suppressed by BCR treatment in both CTRL and CM-KO PP mice (Fig. 4E). However, apoptotic cell subpopulations were suppressed by BCR treatment in CTRL but not in CM-KO PP mice (Fig. 4F). These results suggest that PERK deletion reversed the cardioprotective effect of BCR during the peripartum term, reducing the levels of TNF and apoptotic cardiomyocytes.

### Effects of 23 K PRL in NP mice

To identify the factors through which BCR suppresses the expression of TNF in CTRL mice during the peripartum period, we treated CTRL or CM-KO NP mice with 23 K PRL for three weeks. Neither of the treatments affected cardiac function (Fig. S2A) or sBP (Fig. S2D). PRL treatment induced cardiac dilatation and cardiac hypertrophy in CM-KO but not in CTRL mice (Fig. S2B,C). The cardiac expression of TNF was advanced by PRL treatment in CM-KO mice and not in CTRL mice (Fig. S2E). These results suggest that 23 K PRL treatment induced cardiac mal-remodeling and the upregulation of TNF in CM-KO but not in CTRL mice.

## Discussion

Multiple signaling pathways and various cytokines have been individually shown to be involved in the pathogenesis of PPCM; however, systematic evaluation of the interactions among them has not been extensively reported. In this study, we comprehensively examined how 23 K PRL-PERK axis modulated PPCM-related signaling pathways, using transcriptomic analysis.

It is known that 23 K PRL protects against hypoxia^5^ or inflammation-induced cell death^17^ through AKT and STAT3 signaling. However, the involvement of UPR or PERK signaling with PRL signaling has not been reported. Our data suggest that 23 K PRL, but not 16 K PRL, activated the PERK signaling both in normoxic and hypoxic NRCMs (Fig. 1A). In CTRL PP mice, the expression of PERK inhibitors, such as *Dnajc3*^11^ and *Rbm3*^12^, was decreased, resulting in the activation of PERK downstream signaling. Additionally, pathway analysis showed that the expression of translation-related genes, such as PERK target genes, was the highest in postpartum hearts (Fig. 3B). PERK signaling involves interaction with the other signaling molecules of UPR, namely IRE1α-XBP1 and ATF6^8^. *PERK* deletion induced PPCM-like phenotype during the postpartum period, suppressing the expression of *Xbp1, Atf6,* and oxidative stress response genes, such as *Stat3 and Sod1* (Fig. 3C). However, acute inhibition of PERK activity reduced processing of 23 k PRL to 16 k PRL (Fig. 1A,C). Thus, consecutive PERK inhibition induces PPCM-like phenotype, but temporary PERK inhibition during the peripartum period may improve the cardiac mal-angiogenesis. If selective PERK inhibitors are developed in future, these could be candidates for new drugs of PPCM.

Furthermore, 16 K PRL is a metabolite of 23 K PRL produced by cathepsin D- and MMPS-mediated cleavage. The expression of *Mmps* was induced by JAK-STAT signaling^18^. The expression of *Mmp 2, Mmp3, Mmp9, Stat1, Stat2,* and *Stat3* was increased in CTRL PP mice, but not in CM-KO PP mice (Fig. 3C). As a result, 16 K PRL was produced in CTRL PP mice, but not in CM-KO PP mice (Fig. 3D). Moreover, 16 K PRL has an antiangiogenic effect; however, cardiac angiogenesis in CTRL PP mice was the most advanced among all groups (Fig. 2F,G). The expression of a pro-angiogenic gene, *Vegfa* (Fig. 2I), was the highest in CTRL PP mice. In PPCM patients, circulating and cardiac PAI-1 expression are up-regulated^13^. Circulating PAI-1 (*Serpine1*) seems add 16 K PRL to induce vascular impairment via the uPAR/NFkB/miR-146a pathway. The expression of *Serpine1*, inhibited by STAT3, was suppressed in CTRL PP mice compared to CM-KO PP mice (Fig. 3C). Additionally, 16 K PRL has a proinflammatory effect in endothelial cells and activates NFκB signaling. We showed that 16 K PRL upregulated the expression of *Nfkb2* in NRCMs (Fig. 1E). Interferon-beta target genes were also upregulated in CTRL PP mice (Fig. 2B). Therefore, vascular impairment induced by 16 K PRL and PAI-1 was not observed in CTRL PP mice, but cardiac inflammation occurred.

PERK-DDIT3 signaling is known to induce apoptosis via ER-associated degradation (ERAD). However, a previous study has shown that inhibition of PERK during ER stress induces ERAD-independent apoptosis^19^. In this study, PERKi did not inhibit the expression of cleaved caspase 3, a marker for apoptosis, in hypoxic NRCMs (Fig. 1A). The expression of *Cycs* was the highest in CM-KO PP mice, whereas that of *Ddit3* was not increased (Fig. 3C).

TNF-TNFRSF1A signaling is a pivotal pathway for apoptosis in cardiomyocytes^20^. Furthermore, the expression of *Tnf* (Fig. 2I) and *Tnfrsf1a* (Fig. 3C) was advanced in CM-KO PP mice compared to CTRL PP mice. A previous study has revealed that 23 K PRL treatment upregulated the expression of *Tnf*, while BCR downregulated it^21^. To examine their effects, we treated PP mice with BCR and NP mice with 23 K PRL. These treatments did not affect the cardiac function in these mice (Fig. 4A, Fig. S2A). BCR inhibited the population of apoptotic cardiomyocytes in CTRL mice, but not in CM-KO mice (Fig. 4F). BCR did not suppress the cardiac TNF level in CM-KO mice (Fig. 4C), while 23 K PRL promoted it (Fig. S2E). Moreover, 23 K PRL induced cardiac mal-remodeling (Fig. S2B,C).

Furthermore, we evaluated the expression of genes involved in PPCM, such as titin (*Ttn*)^22^ and cardiac troponin T (*Tnnt2*)^23^. RNA-seq revealed that the expression levels of *Ttn* and *Tnnt2* was the highest in CTRL PP mice compared to the others. Moreover, the expression of *Rbm20*, which controls titin (*Ttn*) splicing^24^, and the expression of *Ttn*-associated genes^25^ was the highest in CM-KO PP mice. TTN and TNNT2 are involved in passive stiffness and contraction of muscle, respectively. Therefore, it is difficult to induce a cardiac muscle stress response during pressure overload without the upregulation of these genes in CM-KO mice.

The expression of *Stat1, Stat2,* and *Stat3* was also increased in the hearts of CTRL PP mice compared to CM-KO PP mice (Fig. 3C). STAT3 is cardioprotective, suppressing the production of ROS by upregulating the expression of antioxidant genes, such as *SOD*^2^. However, the roles of STAT1 and STAT2 in postpartum hearts remain to be uncovered and need to be investigated in the future.

**Figure 5:**
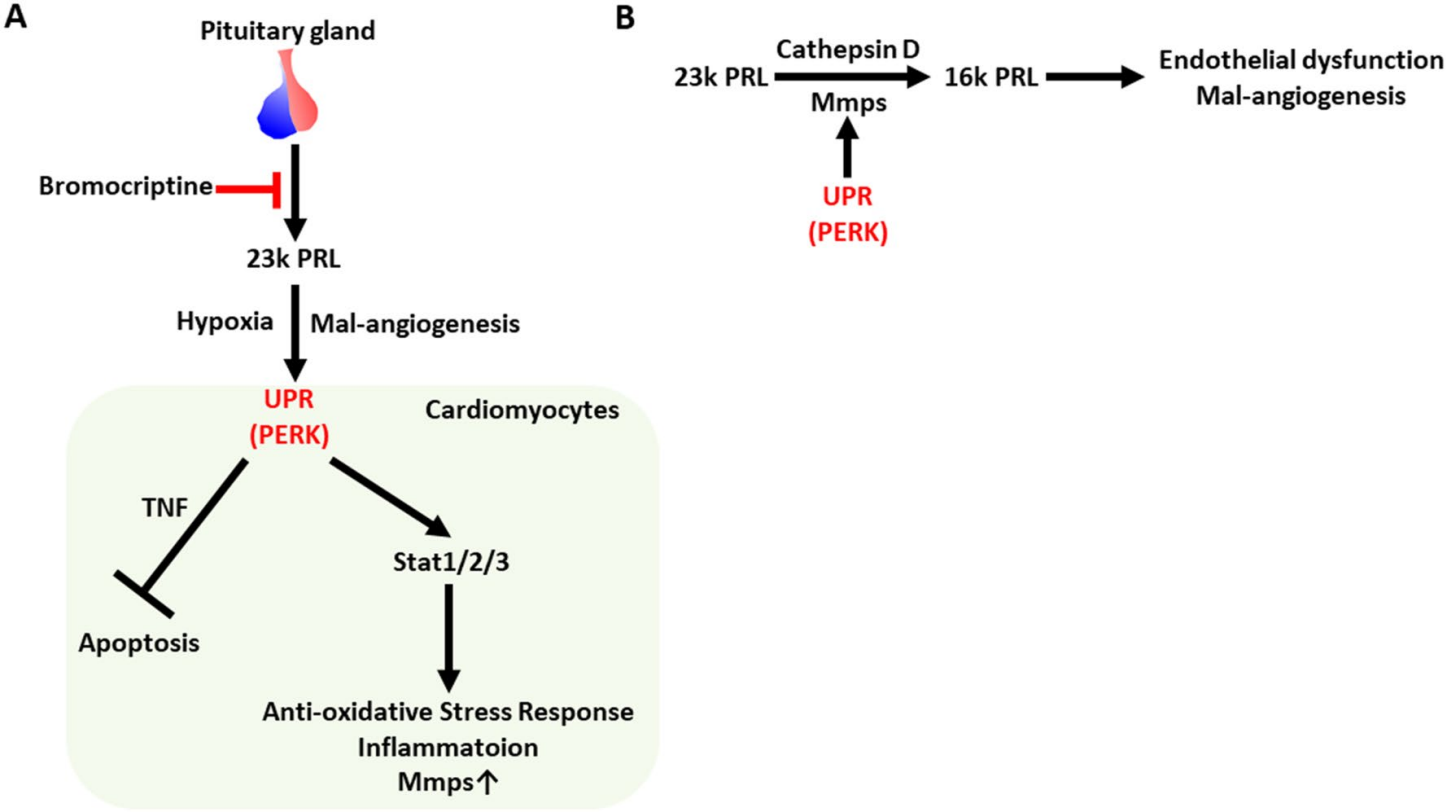
Proposed mechanism of postpartum cardiomyopathy-like cardiac remodeling in CM-KO mice. (**A**) Bromocriptine is known to suppress the release of 23 k PRL from pituitary gland. Various ER stress inducers, such as hypoxia and inflammation, were occurred in the maternal body during the peripartum term. Cardiac PERK deletion promotes apoptosis induced by TNF, while inhibits Stat1/2/3 downstream signalings, such as anti-oxidative stress response, inflammation and the upregulation of Mmps. (**B**) The expressions of Mmps, enzymes for cleavage of 23 k PRL into 16 k PRL, were inhibited during the postpartum period by cardiac PERK deletion.

In conclusion, our findings suggest that peripartum 23 K PRL-PERK interaction is cardioprotective, involving the downregulation of apoptosis and 16 K PRL production and the upregulation of STAT3 signaling (Fig. 5A,B).

## Materials and methods

### Ethics statement

All experiments were approved by the University of Tokyo Ethics Committee for Animal Experiments (P18-097) and strictly adhered to the guidelines for animal experimentation of the University of Tokyo. This study was carried out in compliance with the ARRIVE guidelines.

### Cells and reagents

Isolation and culture of primary rat neonate cardiomyocytes (NRCMs) was performed as described^26^. NRCMs placed in a humidified incubator set at 37 °C in either normoxic conditions (21% O2) or hypoxic conditions (4% O2) with or without rat recombinant PRL (50 nM ProSpec) or PERK inhibitor (gsk2606414, 1 μM, Medkoo) for 24 h (h). NRCMs were also transfected with 23 k PRL plasmid (addgene #73123) or 16 k plasmid, which is mutated from 23 k PRL plasmid, using KOD plus mutagenesis kit (Toyobo). siRNAs and plasmids were transfected using Lipofectamine 3000 (Thermo Fisher Scientific) according to the manufacturer’s protocol.

### Mice

To generate mice with cardiomyocyte-specific deletion of PERK mice homozygous for the floxed PERK (The Jackson Laboratory, Bar Harbor, ME) were crossed with αMHC-Cre mice. Mice were maintained on a standard rodent chow diet with 12-h light and dark cycles. Mice were bred starting at the age of 8 weeks. Administration of bromocriptine (4 mg/kg/day, Cayman Chemical) was added in drinking water. For chronic (3 week) administration, osmotic minipumps (Alzet; LEP or PRL 400 iU/kg/day) were implanted in sedated nulliparous mice.

Transthoracic echocardiography was performed with a VEVO2100 (Visualsonics, Toronto, Canada) system in nonanesthetized mice. Parasternal short-axis projections were visualized and M-mode recordings at the mid-ventricular level were recorded. Heart rate, left ventricular (LV) end-systolic and end-diastolic dimensions were averaged from 5 beats. LV percentage of fractional shortening (FS) was then calculated %FS = 100 × (LVEDD–LVESD)/LVEDD. Systolic blood pressure (sBP), diastolic blood pressure (dBP), and mean blood pressure (mBP) were measured by tail-cuff plethysmography from Muromachi Kikai Co., Ltd. (Tokyo, Japan) before mice were killed. Rectal body temperature was measured by thermometer.

### Isolation of cardiomyocytes from adult mice

The isolation and purification of cardiomyocytes from the adult mice were performed as described before^1^. Briefly, the cardiacmyocytes were isolated from mouse hearts via coronary perfusion with collagenase type 2 (Worthington).

### mRNA isolation, cDNA synthesis and qRT-PCR

mRNA from tissue or cells was isolated with the Trizol or miRNeasy Mini Kit (Qiagen). cDNA synthesis was made with High Capacity cDNA Reverse Transcription Kits (Thermo Fisher Scientific) and quantitative PCR was performed with THUNDERBIRD SYBR qPCR Mix (TOYOBO, #QPS-201), using LightCycler480 (Roche Applied Science) or QuantStudio™ 5 Real-Time PCR System (Thermo Fisher Scientific). GAPDH was used as a loading control.　PCR primers are these; rat ATF4 Forward: 5′-CTACTAGGTACCGCCAGAAG-3′, Reverse:5′-GCCTTACGGACCTCTTCTAT-3′, rat GAPDH Forward 5′-GACATGCCGCCTGGAGAAAC-3′, Reverse 5′-AGCCCAGGATGCCCTTTAGT-3′, mouse TNF Forward 5′-ACAAGGCTGCCCCGACTAC-3′, Reverse 5′-TCTCCTGGTATGAGATAGCA-3′, mouse ErbB4 Forward 5′-AATGCTGATGGTGGCAAGA-3′, Reverse 5′-CATCACTTTGATGTGTGAATTTCC-3′, mouse VEGFA Forward 5′-AAAAACGAAAGCGCAAGAAA-3′, Reverse 5′-TTTCTCCGCTCTGAACAAGG-3′, mouse GAPDH Forward 5′-CATGGCCTTCCGTGTTCCTA-3′, Reverse 5′-CCTGCTTCACCACCTTCTTGAT-3′.

### RNA-seq library preparation

Total RNAs from NRCMs and hearts were isolated as described above. The RNA integrity score was calculated with the RNA 6000 Nano reagent (Agilent Technologies) in a 2100 Bioanalyzer (Agilent Technologies). RNA-Seq libraries were prepared with a TruSeq RNA Library Prep Kit (Illumina). The libraries were sequenced on a HiSeq 2500 system (Illumina) as single read 100 (NRCMs) or 150 (hearts) base reads.

### RNA-seq data analysis

Sequence reads were aligned to the rat or mouse reference genome (RGSC6.0/rn6 or GRCm38/mm10) with HISAT2 (version: 2.1.0) with the default parameters. After enumerating the mapped reads on the gene positions deposited in the geocode database (https://www.gencodegenes.org/mouse/), the FPKMs (fragments per kilobase of exon per million reads) of all of the deposited genes were calculated by CuffLinks with the default parameters. RNA-seq signals were visualized with the Integrated Genome Viewer (Version 2.4.8) (http://software.broadinstitute.org/software/igv/). The RNA-seq signal of each locus was normalized by the following basis:

$$ Signal\;of\;each\;locus = \frac{Number\;of\;mapped\;reads\;on\;each\;locus \times 1,000,000}{{Total\;number\;of\;mapped\;reads}} $$

### Reproducibility between RNA-seq experiments

The reproducibility of the genome-wide RNA-seq signals in the biological replicates was examined under all conditions. The FPKM values were used as the RNA-seq signals. Subsequently, the correlation coefficients between three biological replicates were calculated based on the FPKMs of each reference gene.

### Volcano plot

To visualize the dispersion of mRNAs, the log2 fold changes (FCs) of expression levels (horizontal axis) and the − log10 *P* values (vertical axis) were plotted.

### Signaling pathway analysis

Gene annotation enrichment analysis was performed for KEGG pathway analysis, using the functional annotation tool in DAVID Bioinformatics Resources 6.8 (http://david.abcc.ncifcrf.gov/), as described. Among them, genes that expressed above 5 reads in one of the replicates in all groups, were considered for analysis.

### Flow cytometry

Dead cells and debris were excluded using forward scatter/side scatter (FSC/SSC). For the analysis of dying cells, FITC labeled Annexin V (1:100 dilution, BioLegend) and 4′,6-diamidino-2-phenylindole (DAPI, 1:1000 dilution, Sigma-Aldrich) can also be used. Necroptotic or apoptotic cardiomyocytes were defined as DAPI^+^/Annexn V^+^, or DAPI^−^/Annexn V^+^ cells, separately.

### Protein aggregation and ROS assays

Protein aggregation was assessed in isolated cardiomyocytes from adult mice, using Proteostat protein aggregation assay kits (Enzo Life Sciences). Proteostat emits fluorescence when it binds to the tertiary structure of aggregated proteins. It has been validated to specifically detect protein aggregates and aggresome-like inclusion bodies in cells and cell lysates. To double-label protein aggregates and α-actinin, tissue sections and cultured cells were incubated with anti-α-actinin antibody (Sigma-aldrich)/secondary antibody-fluorescein (488 nm) in PBS and Proteostat dye in Enzo assay buffer for 30 min at room temperature, followed by observation under a fluorescent microscope and image acquisition.

Cytoplasmic protein aggregation or inclusion body formation in isolated cardiomyocytes was analyzed as described^4^. Briefly, isolated cardiomyocytes were fixed with 4% PFA for 15 min. At the end of fixation, cells were washed with PBS and incubated at room temperature for 15 min with permeabilization buffer. Cells were then incubated in dual-color detection buffer for 30 min, and fluorescence was measured using the fluorescence plate reader with 485 nm excitation and 590 nm emission filters for aggresome readings and 355 nm excitation and 460 nm emission filters for Hoechst nuclear readings. Increases in the ratio of the ProteoStat aggresome signal (485/590 nm), relative to the Hoechst signal (355/460 nm), indicate the formation of aggregated proteins within aggresomes and related inclusion bodies.

To determine the expression of ROS in isolated adult cardiomyocytes, we used ROS Fluorometric Assay Kit (Elabscience).

### Capillary density and cardiomyocyte cross-sectional area

Capillary density was determined as the ratio of capillaries to 100 cardiomyocytes in transversely sectioned LV tissue immunostained with isolectin B4 (Vector) and counterstained with tetramethyl rhodamine isothiocyanate-conjugated wheat-germ agglutinin (WGA) and Hoechst^2^. We measure d cardiomyocyte cross-sectional area after staining WGA^5^.

### CD activity assay

Supernatant from freshly isolated LV tissue was generated by mincing LV tissue. Cathepsin D (CD) activity was determined in LV supernatant by using the Cathepsin D Activity Assay Kit (Raybio).

### Western blotting

Mouse tissue or cultured NRCMs were homogenized in lysis buffer (cell signaling technology, #9803) with 1 mM phenylmethylsulfonyl fluoride (PMSF), and lysates were separated on 5–20% polyacrylamide gels and transferred to polyvinylidene difluoride membranes. After blocking with 5% skim milk, the membranes were probed with one of the following primary antibodies overnight at 4 °C: anti PERK antibody (1:200, Santa cruz), anti p-PERK (Thr980) antibody (1:100, cell signaling technology), anti eIF2α antibody (1:1000, cell signaling technology), anti p-eIF2α (Ser51) antibody (1:1000, cell signaling technology), anti PRL (N-terminal) antibody (1:1000, Acris Antibodies GmbH), anti phosphorylated STAT3 antibody (1:2000, cell signaling technology), anti STAT3 antibody (1:1000, cell signaling technology), anti cleaved caspase3 antibody (1:1000, cell signaling technology), anti GAPDH antibody (1:1000, cell signaling technology), and anti transferrin antibody (1:1000, Santa cruz).

This was followed by a 1-h incubation with secondary antibodies conjugated with horseradish peroxidase (HRP) at room temperature. Bound antibodies were detected by chemiluminescence with the ECL detection system. Relative protein levels were quantified using the Image J program (NIH, Bethesda, MD). Some of images (Figs. 1A,F, 3D) did not include full length membranes, with membrane edges visible.

### Statistical analysis

The data are described as mean ± SD. Parametric tests were used after verification to ensure the variables in each group were normally distributed. Student’s un-paired t tests, as well as one-way or two-way analysis of variance (ANOVA) with Bonferroni correction were performed using the Graph Pad Prism8.4.2. The clustering displayed in the heatmap was also performed using this software.

## Supplementary Information

Supplementary Information.

Supplementary Table 1.

Supplementary Table 2.

## Data Availability

The RNA sequence data can be accessed through the Gene Expression Omnibus (GEO) under the NCBI accession number GSE168536 and GSE169063. All other data needed to evaluate the conclusions in the paper are present in the paper or the Supplementary Materials.
