# Supplementary material for: Inhibition of cardiac PERK signaling promotes peripartum cardiac dysfunction
Source: Sci Rep. 2021 Sep 21;11:18687. doi: 10.1038/s41598-021-98344-7 (PMC8455649; doi:10.1038/s41598-021-98344-7)
Supplement: Supplementary file 1 — Supplementary Information. [file 41598_2021_98344_MOESM1_ESM.pdf]

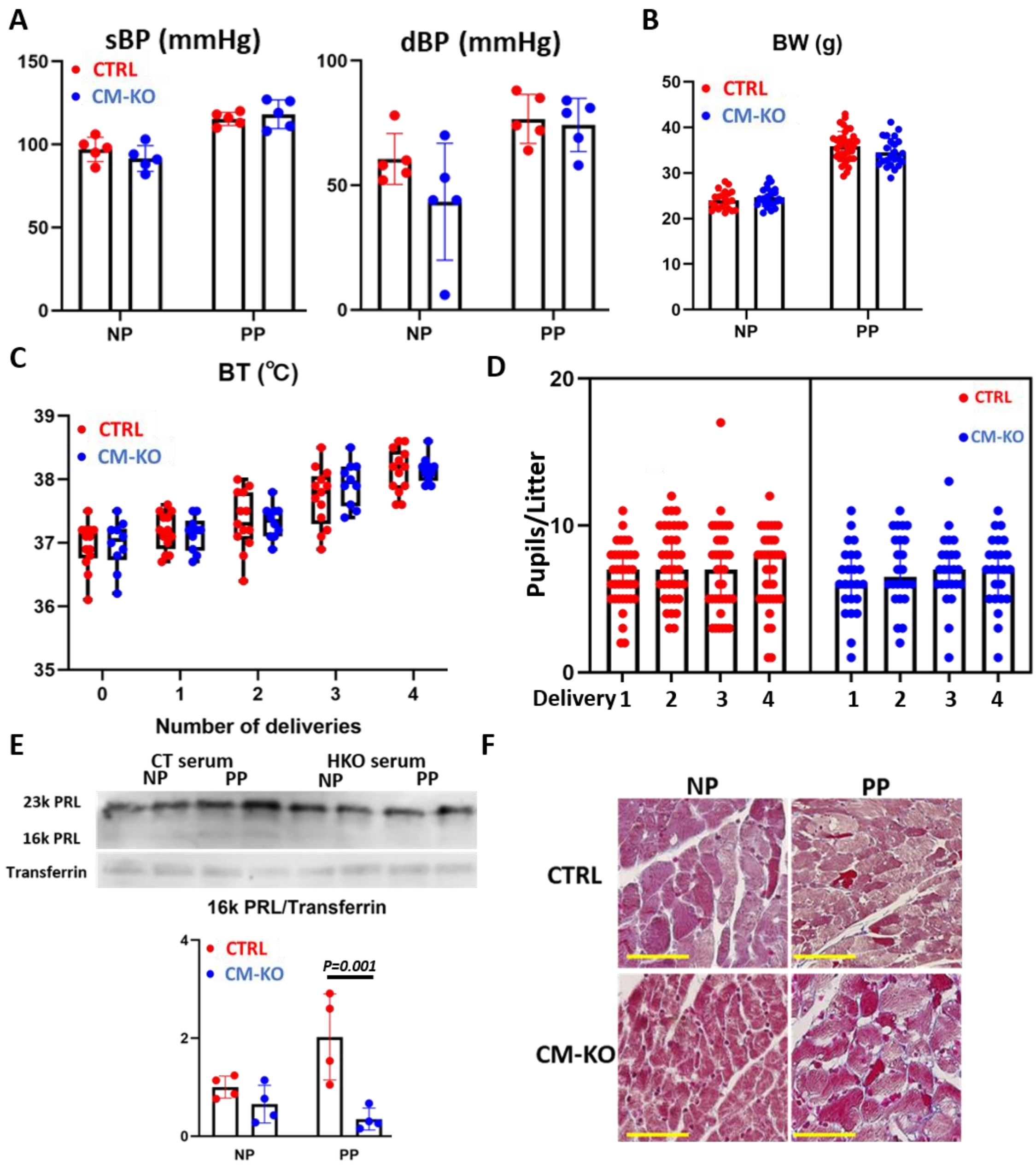

Fig S1. *PERK* deletion did not affect blood pressure, body weight, body temperature, pupils per litter, and cardiac fibrosis in neither nulliparous nor postpartum mice.

(A) Systolic and diastolic blood pressure (sBP and dBP) were not different in CTRL and CM-KO mice, under NP and PP conditions. n=5 mice per group. Data represent mean±SD; P-values were measured by two-way ANOVA with Bonferroni

correction.

(B) Body weight (BW) was not different in CTRL and CM-KO mice, under NP and PP conditions. n=22-37 mice per group. Data represent mean±SD; P-values were measured by two-way ANOVA with Bonferroni correction.

(C) Body temperature (BT) was not different in CTRL (n=17) and CM-KO (n=12) mice, from nulliparous to 4 deliveries. Data represent mean±SD; P-values were measured by two-way ANOVA with Bonferroni correction.

(D) Pupils per litter in each delivery were not different in CTRL (n=37) and CM-KO (n=24) mice. Data represent mean±SD; P-values were measured by nested t-test.

(E) Western blot analysis of prolactin (23k PRL, 16k PRL) and transferrin (loading control) in the sera of NP CTRL, PP CTRL, NP CM-KO, and PP CM-KO mice. Densitometric quantification of the 16k PRL to transferrin protein ratio (n=4 mice per group). Data represent mean±SD; P-values were measured by two-way ANOVA with Bonferroni correction.

(F) Azan staining in hearts of NP CTRL, PP CTRL, NP CM-KO, and PP CM-KO mice. Scale bars: 50 μm.

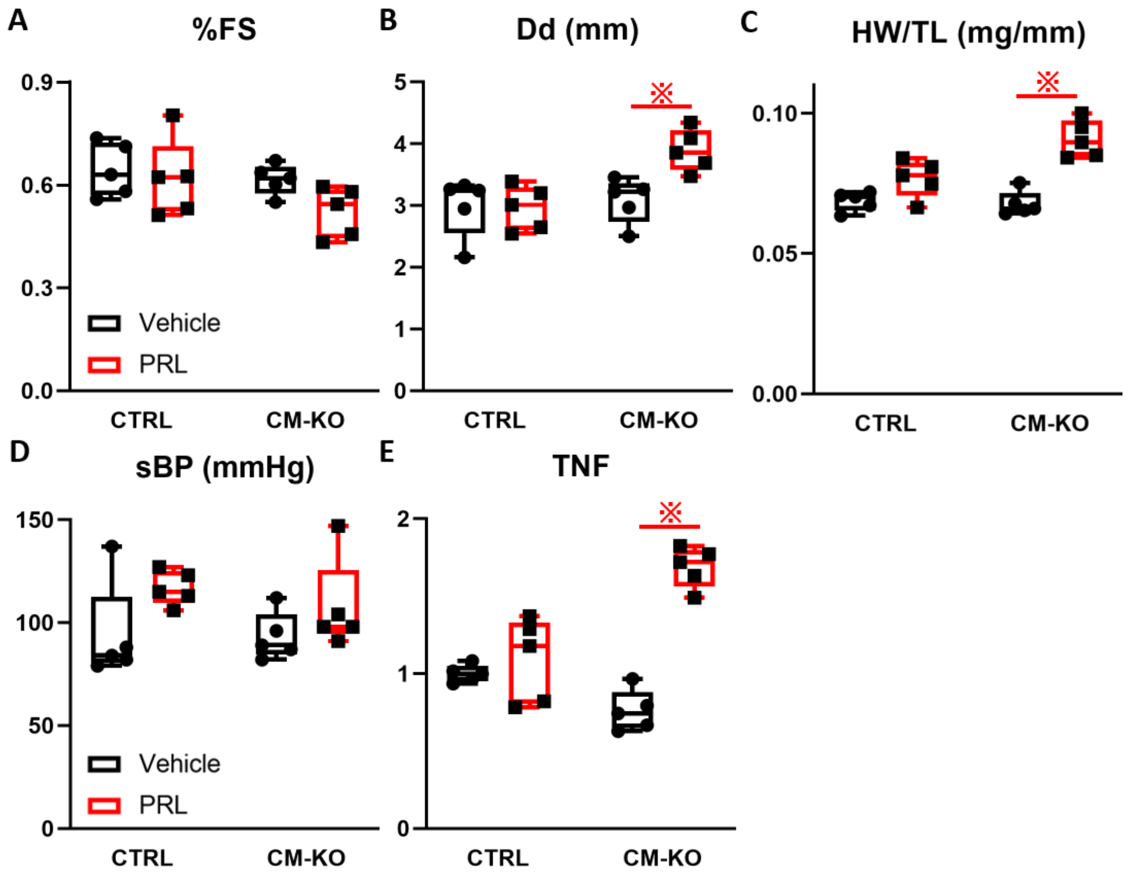

Fig. S2. Administration of 23k prolactin induces cardiac mal-remodeling in CM-KO mice

Effect of 23k prolactin (PRL) administration for 3 weeks on %FS (A), Dd (B), HW/TL (C), sBP (D), and the cardiac TNF levels (E) (n = 5 mice in each group).

Data represent mean ± SD; ※$P<0.05$ two-way ANOVA with Bonferroni correction.

>1. Please ensure the abstract in your manuscript file exactly matches the abstract on our system

I ensure the abstract.

>2. In-house Editorial Comments:

• We note that some of the images (Figure 1A ,1F, 3D, and S1E) are heavily cropped. We ask that as full as possible length gels and blots are included in the Supplementary Information file. These images should be the original, unprocessed versions.

I show bolts in Figure 1A ,1F, 3D, and S1E as these;

Figure 1A blots

・p-PERK(biological replicate1)

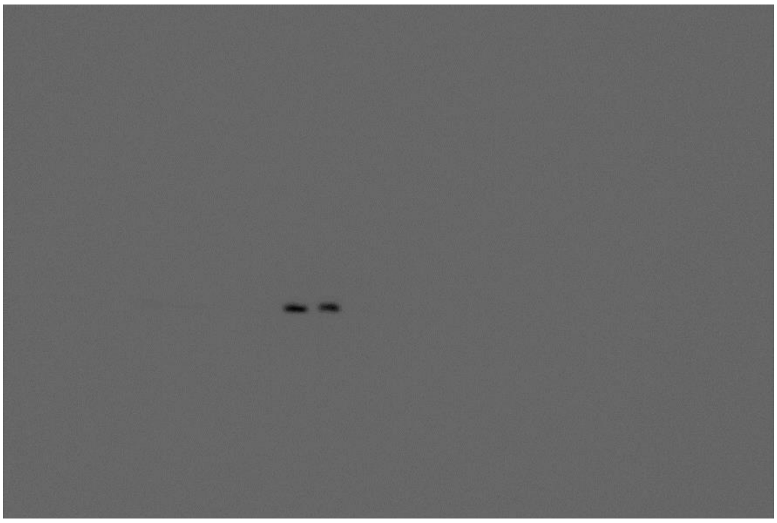

・p-PERK(biological replicate2)

・PERK (biological replicate1)

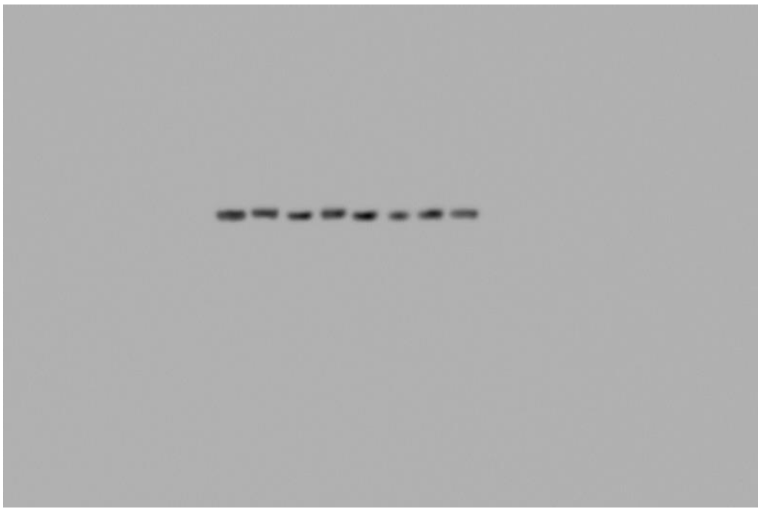

・PERK (biological replicate2)

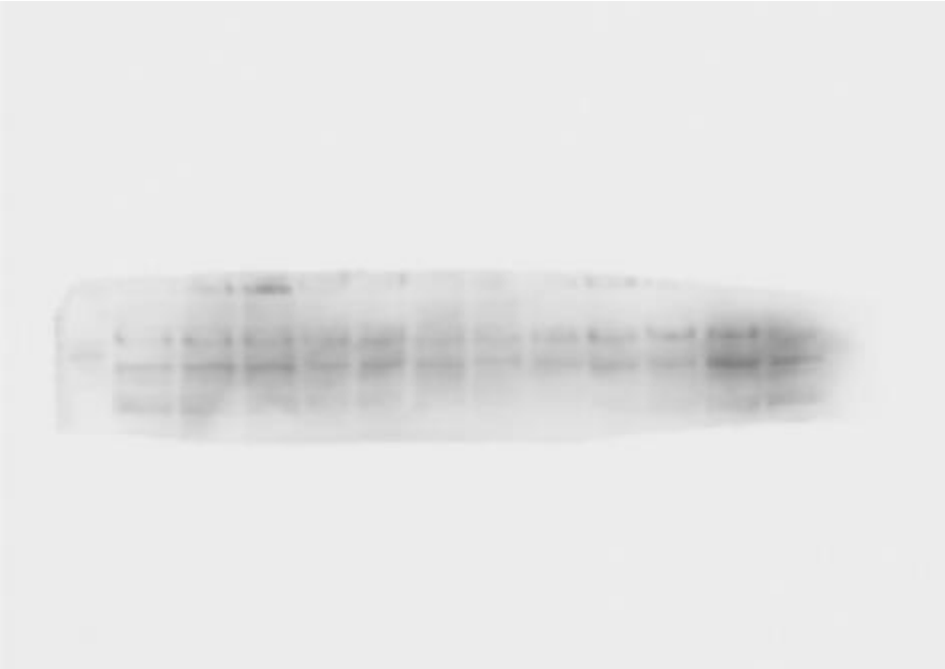

・23k PRL/16k PRL(left 2 lanes were 23k PRL positive controls)

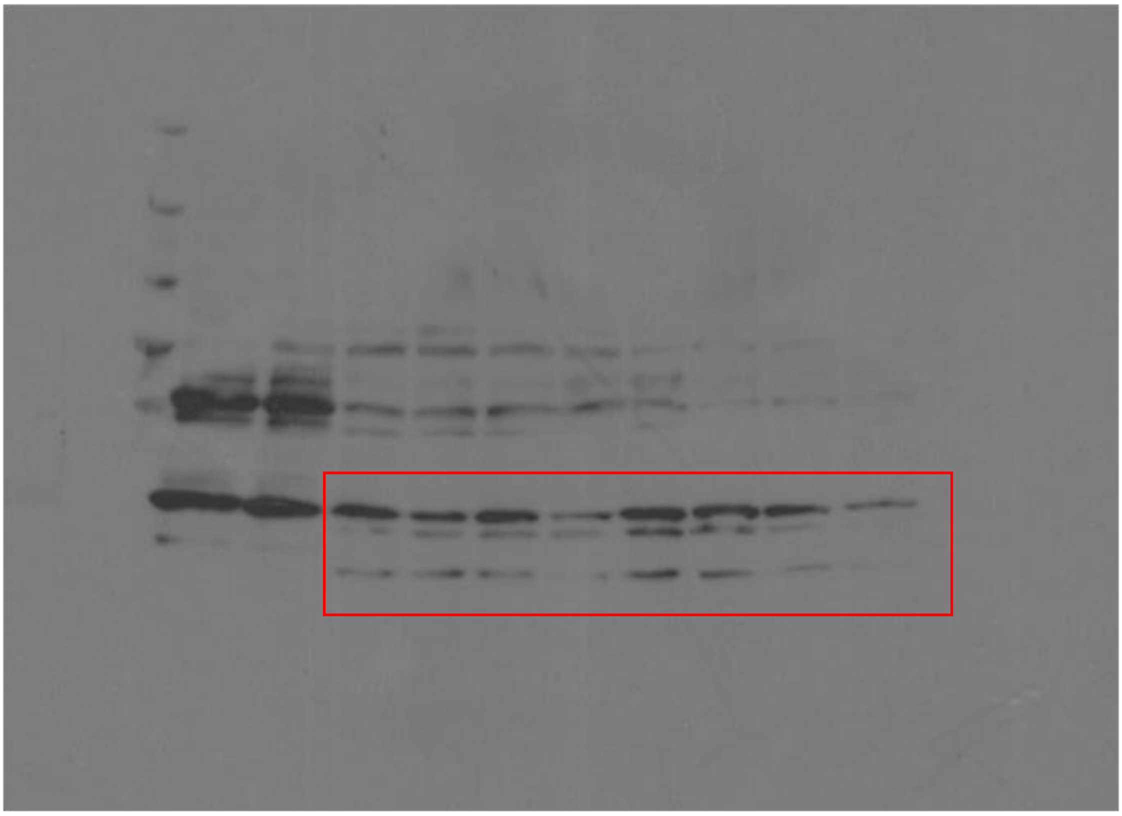

・cleaved caspase3((left and right 2 lanes were negative controls)

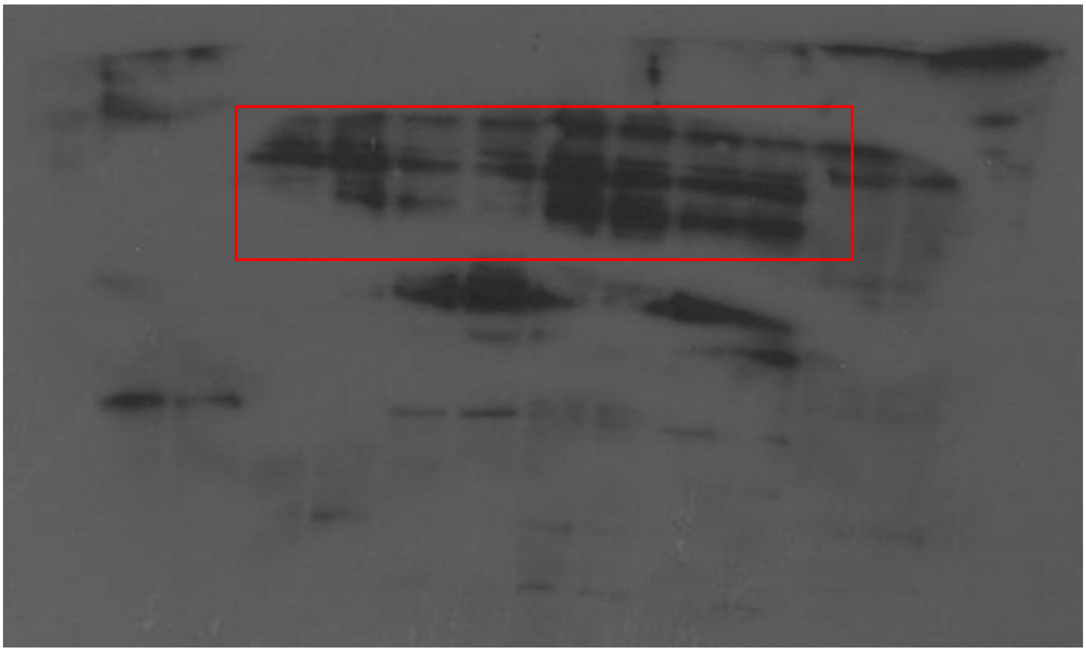

・GAPDH(biological replicate1)

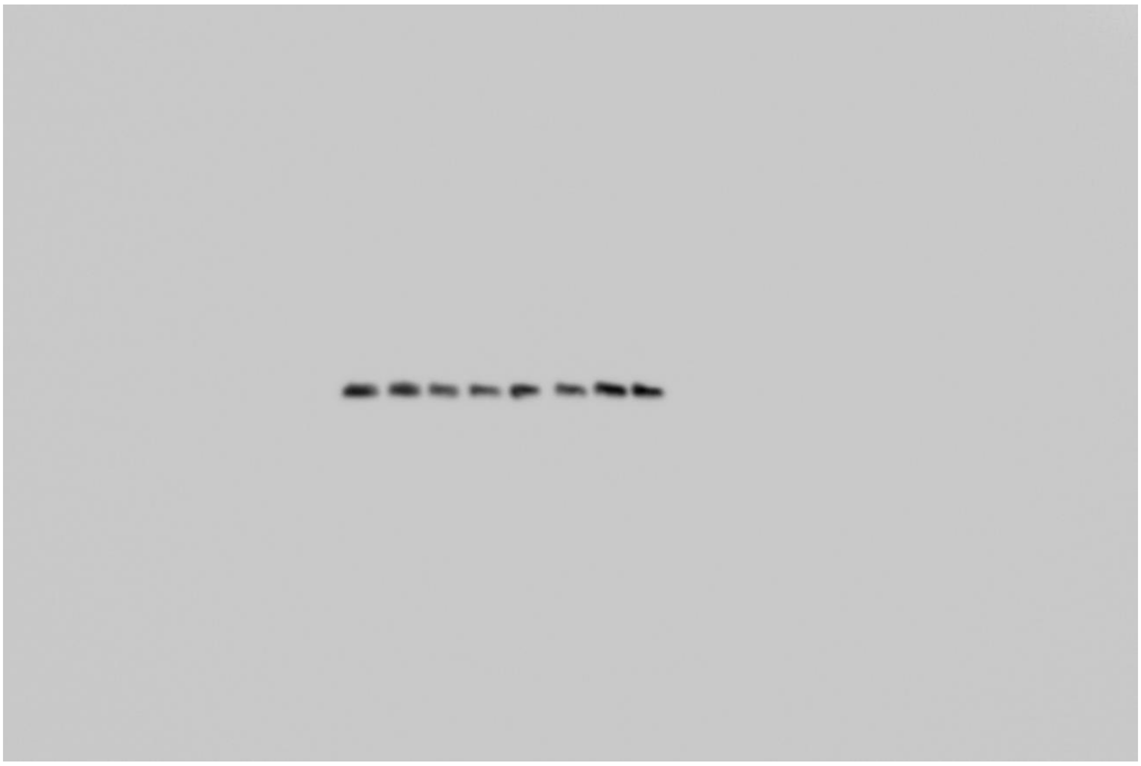

・GAPDH(biological replicate2)

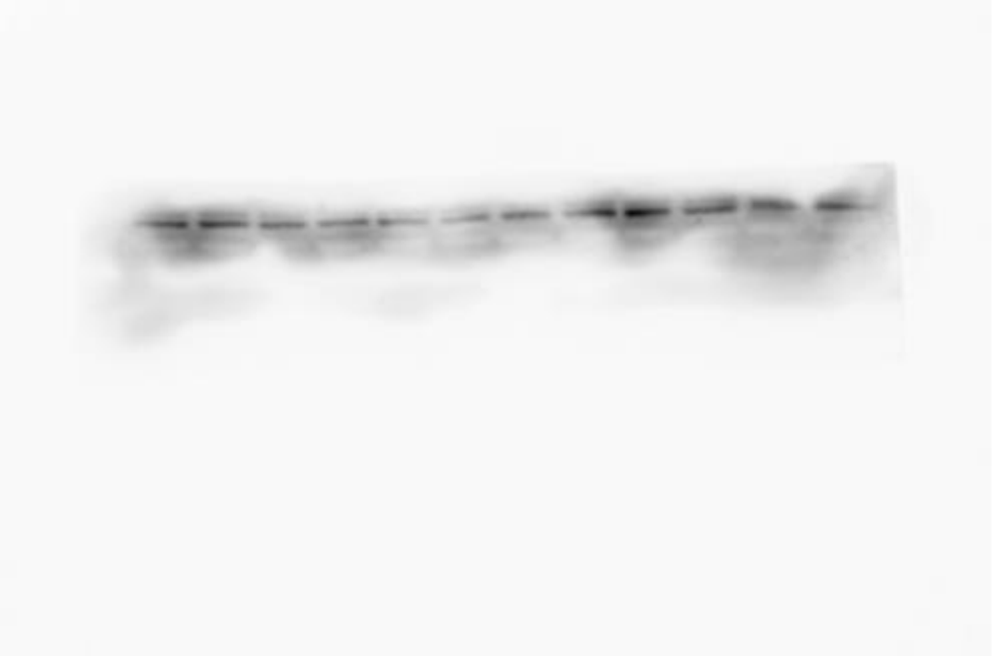

Figure 1F blots

・p-PERK(left and right reversed)

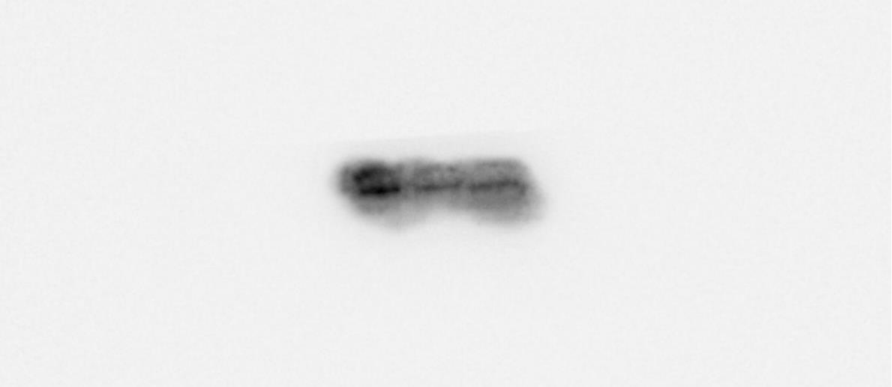

・PERK(left and right reversed)

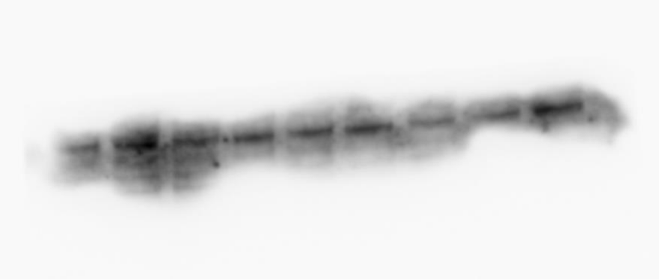

・p-eIF2a(left and right reversed, exposure1)

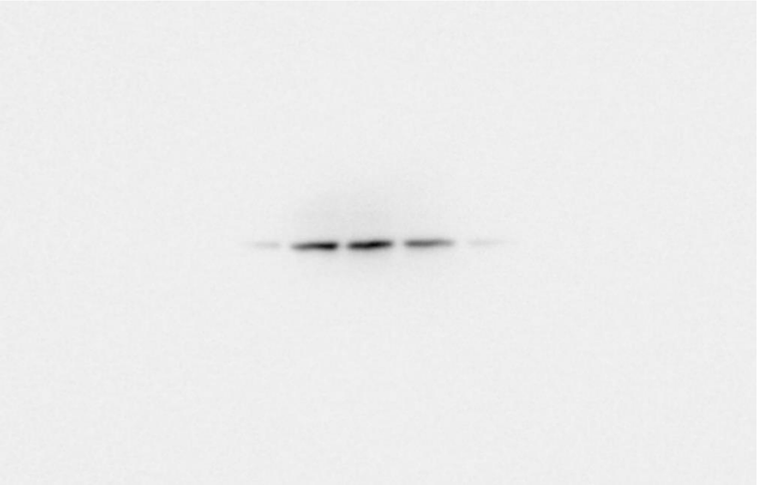

・p-eIF2a(left and right reversed, exposure2)

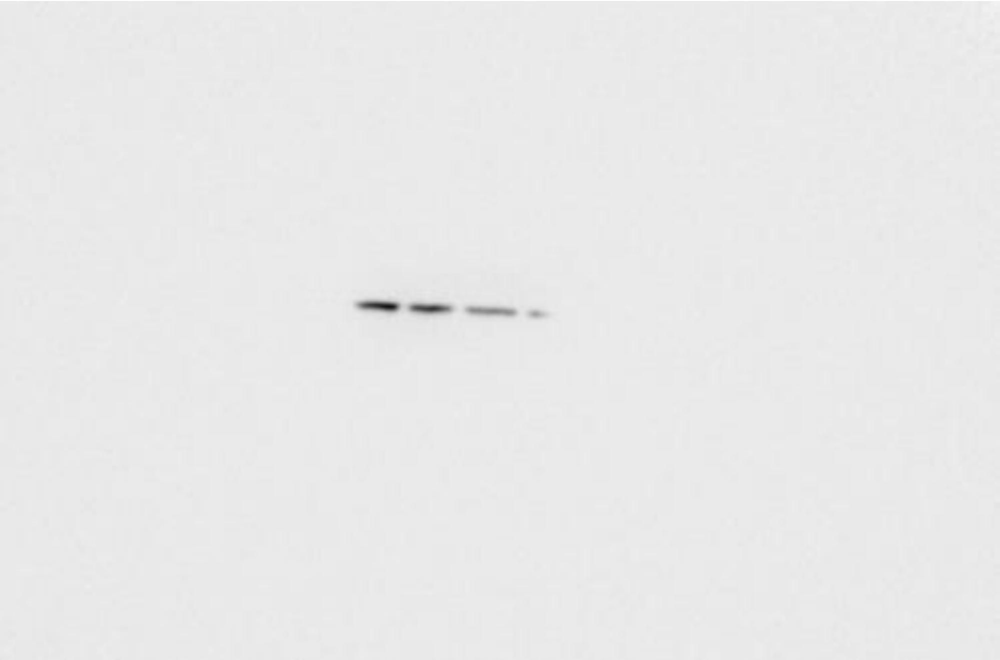

・eIF2a(left and right reversed)

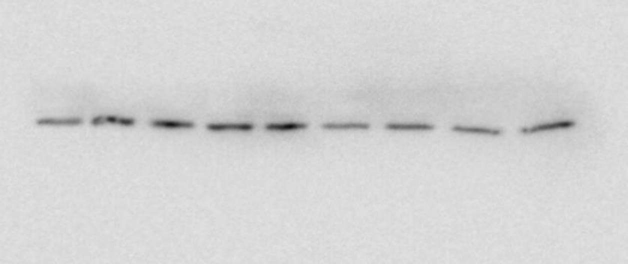

・PRL(left and right reversed)

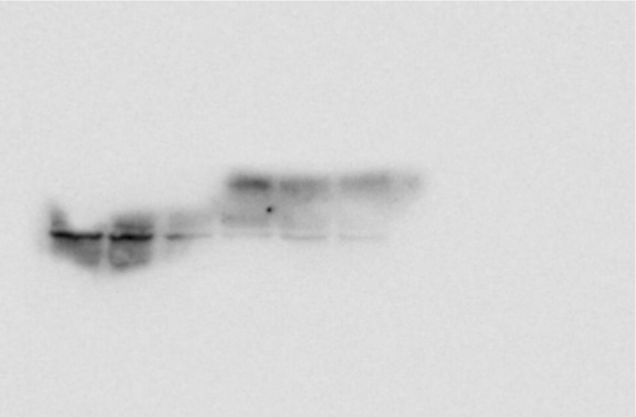

・GAPDH(exposure1)

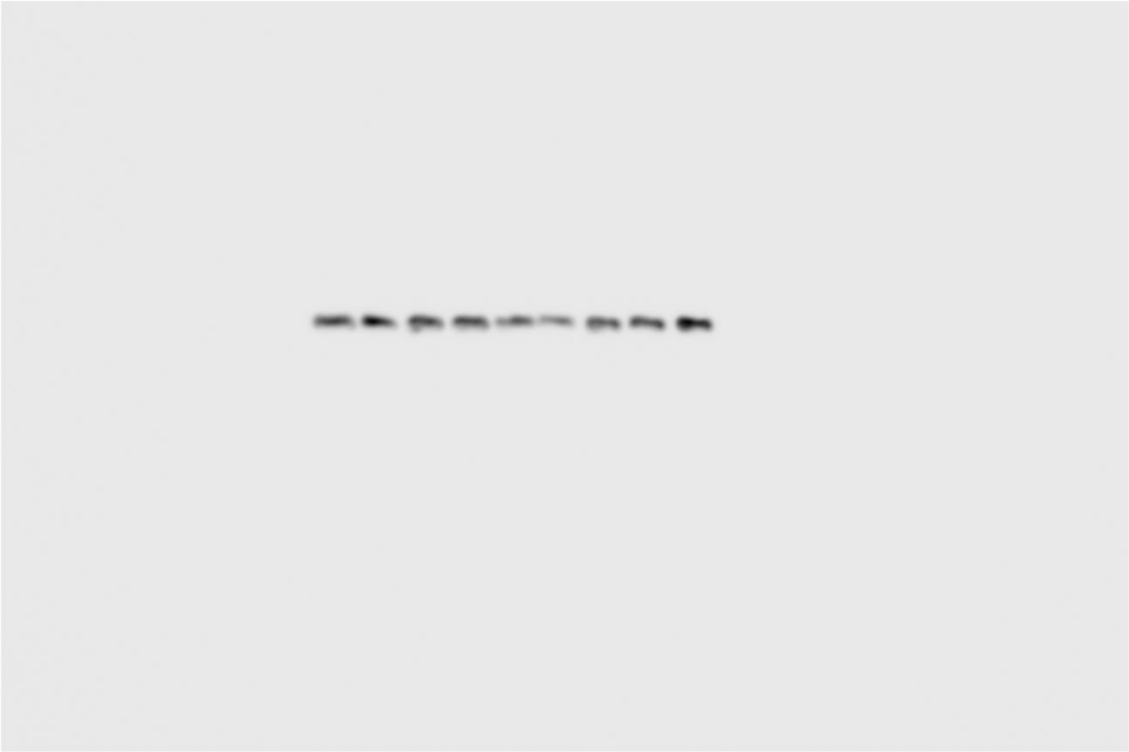

・GAPDH(exposure2)

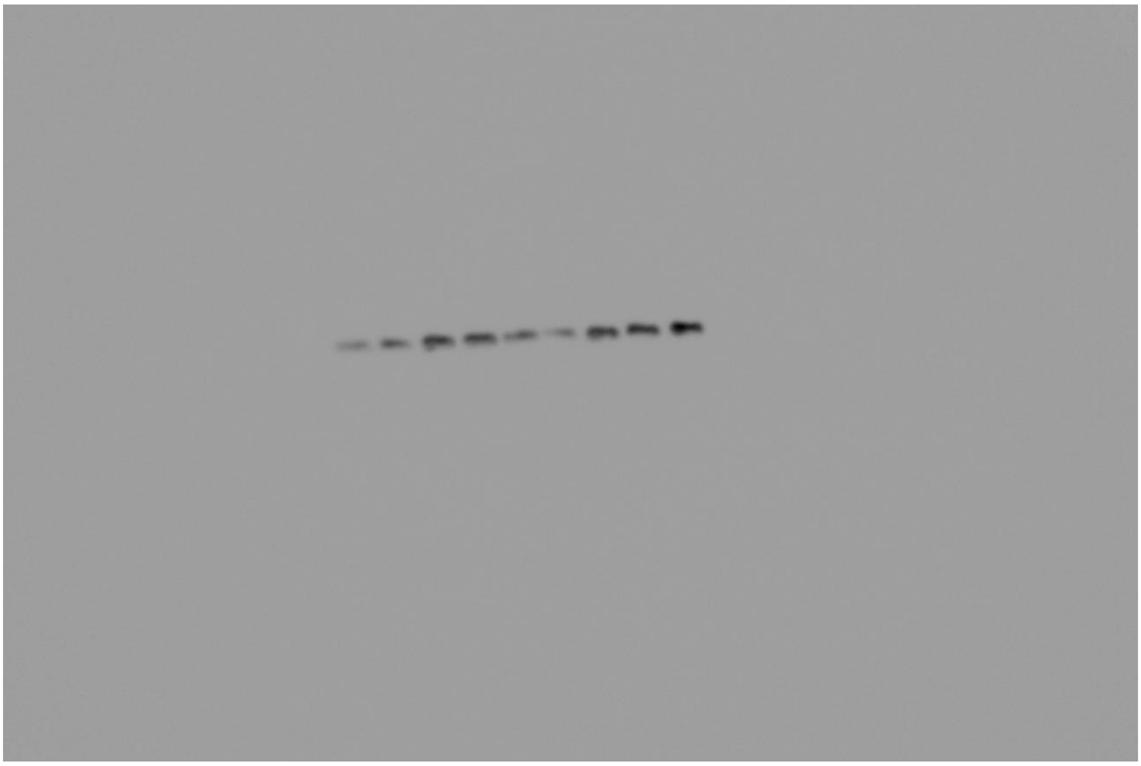

Figure 3D blots

・p-PERK(biological replicate 1)

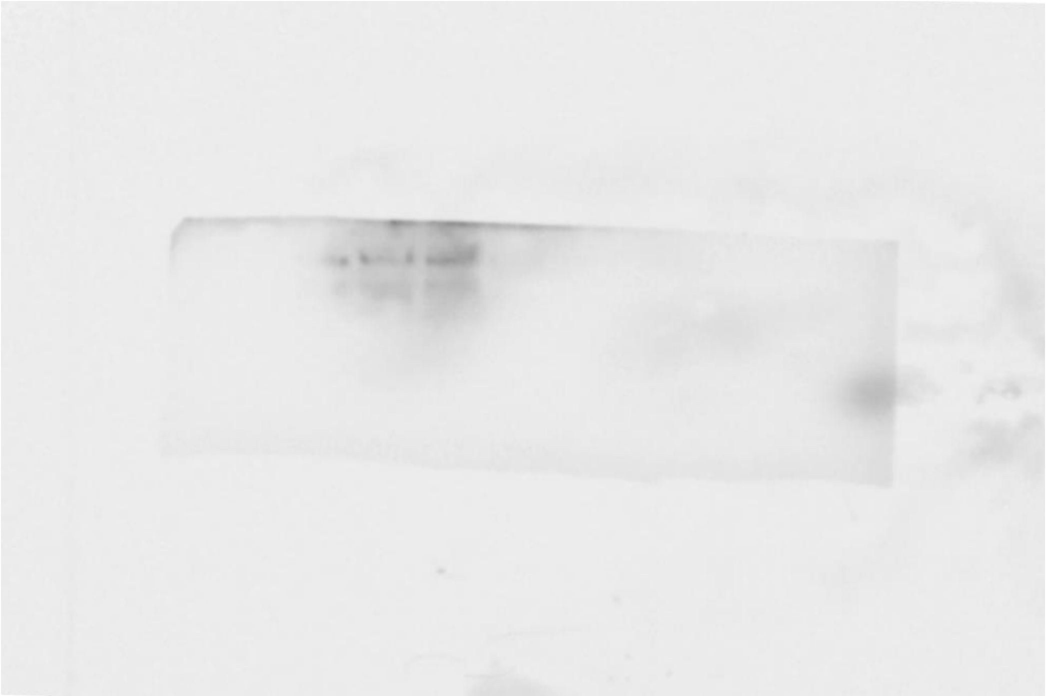

・p-PERK(biological replicate 2)

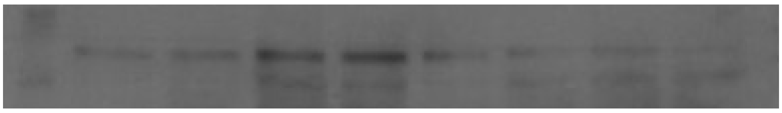

・PERK(biological replicate 1)

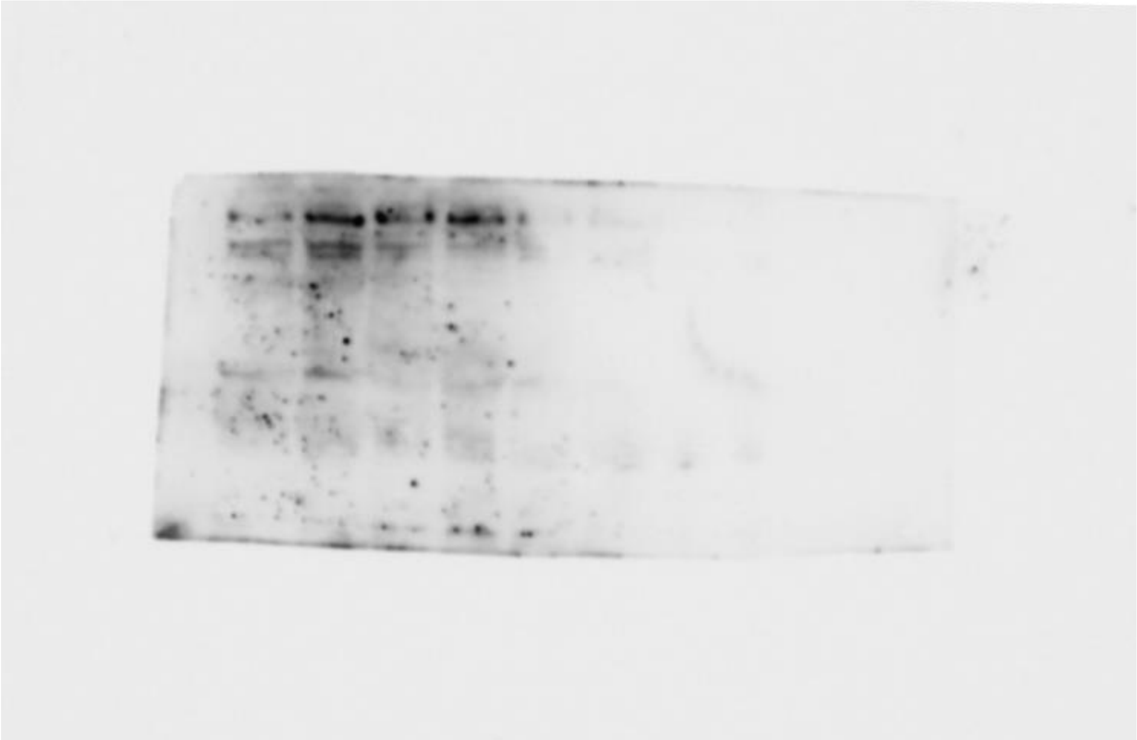

・PERK(biological replicate 2)

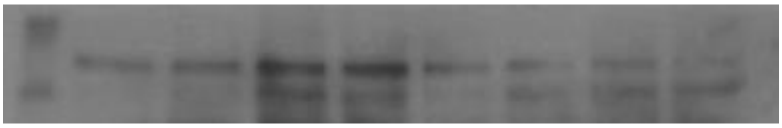

・p-eIF2a(left and right reversed, left two lanes were negative control)

・eIF2a(left and right reversed, left two lanes were negative control)

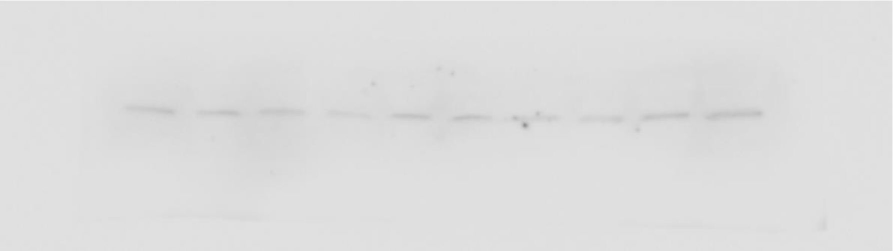

・p-STAT3(left and right reversed, left two lanes were negative control)

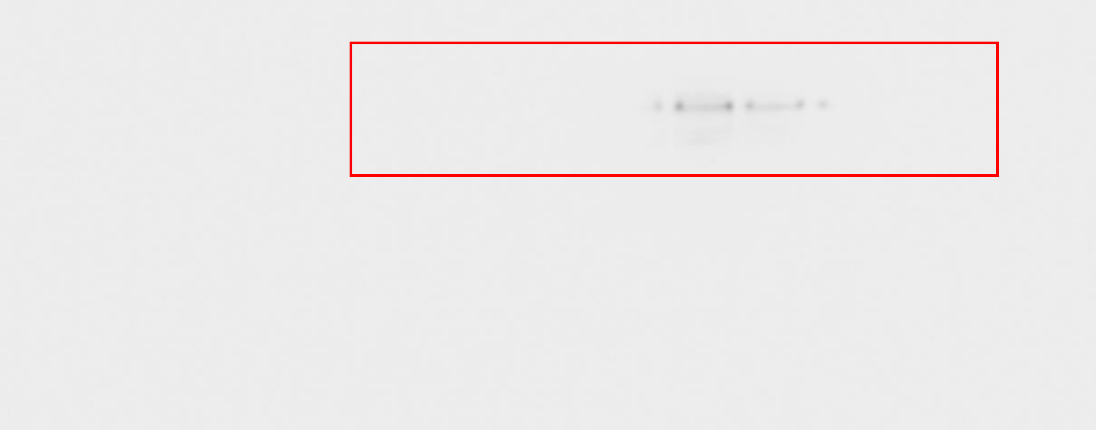

・STAT3(left and right reversed, left two lanes were negative control)

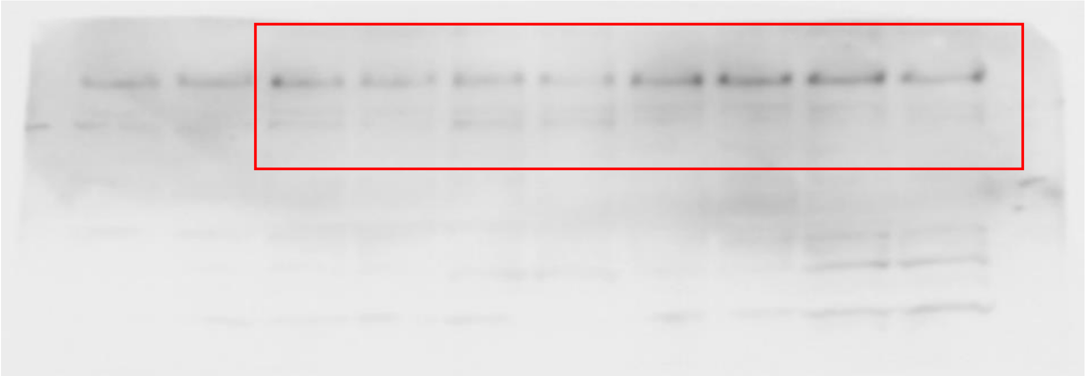

・p-Akt(left and right reversed, left two lanes were negative control)

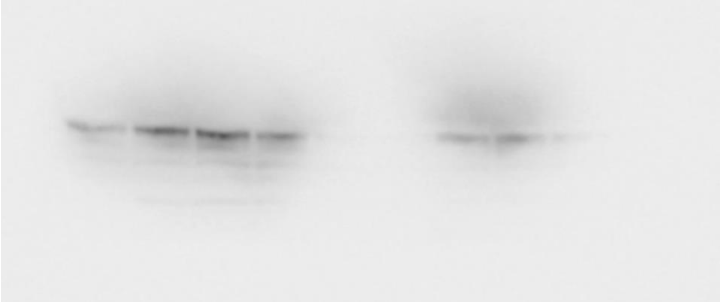

・Akt(left and right reversed, left two lanes were negative control)

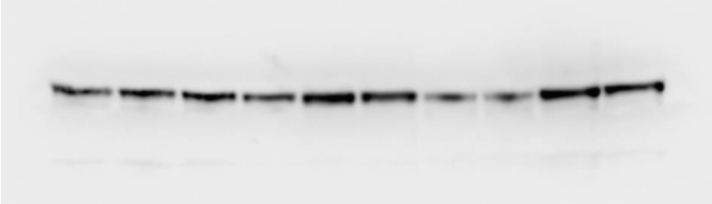

・PRL(left and right reversed)

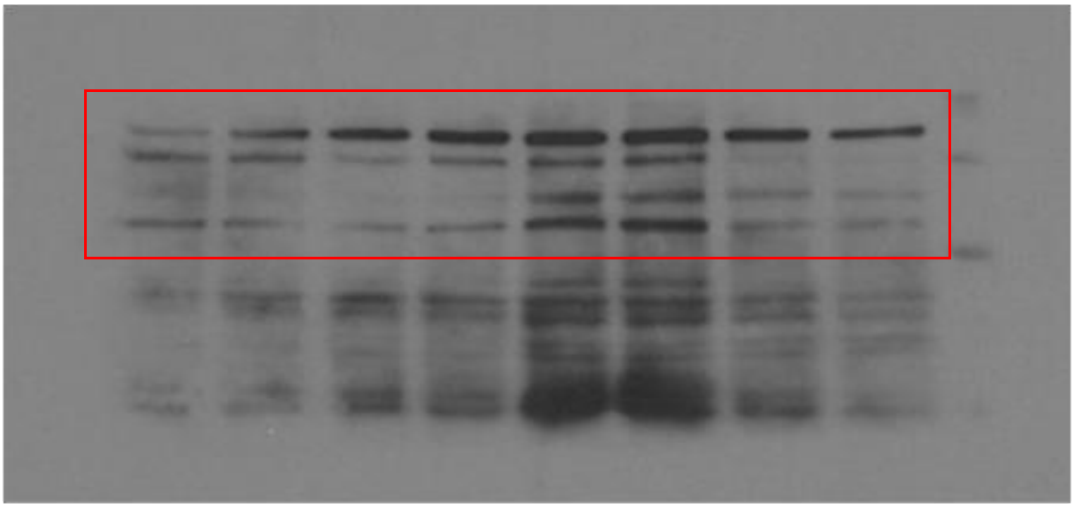

・GAPDH(exposure1)

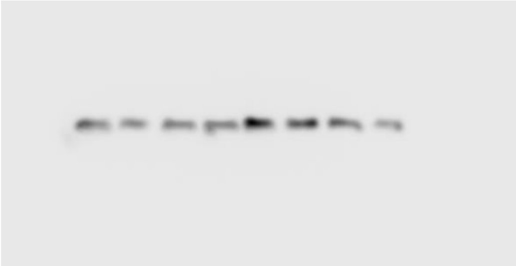

・GAPDH(exposure2)

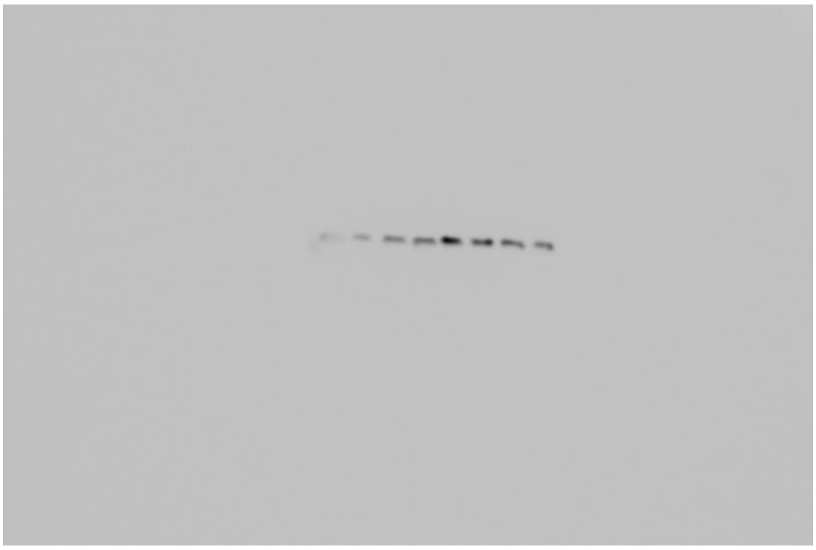

Figure S1E blots

・PRL(left and right reversed, left two lanes were negative control)

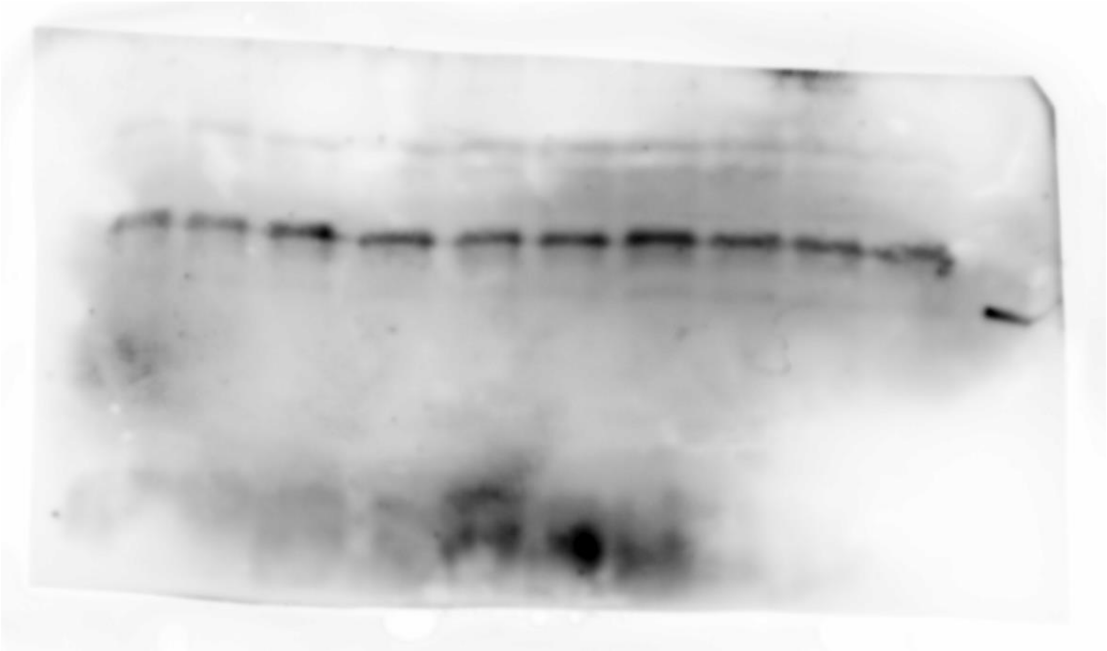

・Transferrin(right two lanes were negative control)

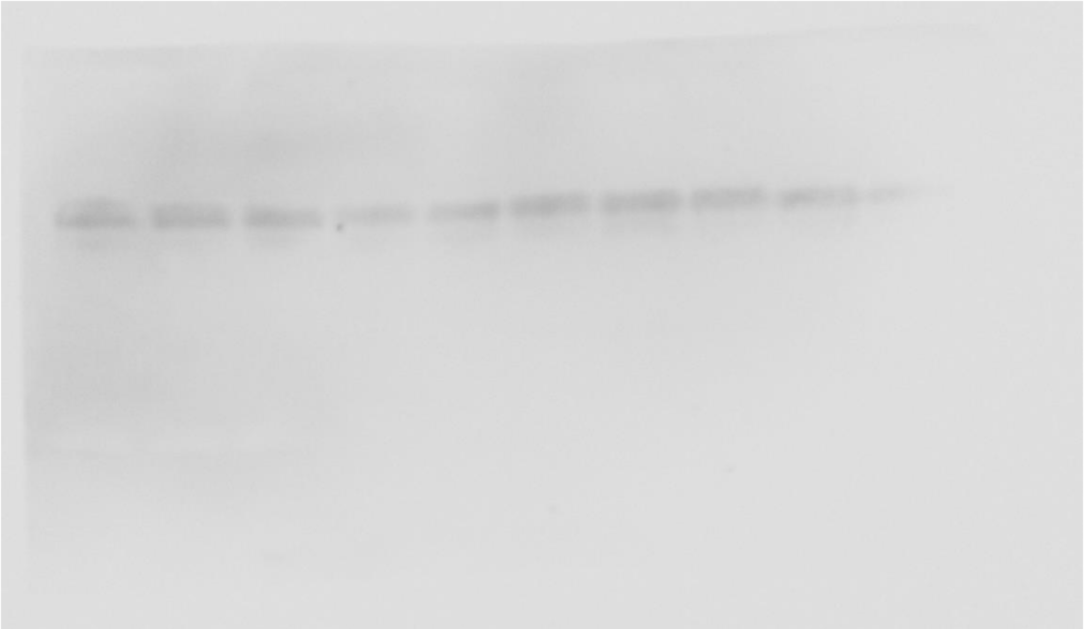

• We note that some of the figures (Figure 1A ,1F, 3D, and S1E) are not accurately delineated. Delineation with dividing lines should be reserved for when non-contiguous lanes from different parts of the same gel, or from different gels, fields, or exposures are grouped together in the figures.

All lanes were contiguous, but some lanes were uneven blotted.

1. Please upload your manuscript file as a Microsoft Word (.doc/.docx) or .tex file. If you wish to upload a .tex file, please ensure that this file is uploaded as a supplementary material file, with a PDF of the file included as the manuscript file.
>I upload manuscript file as a Microsoft Word.

2. Where gels/blots are used in figures authors should check and ensure their compliance with the digital image and integrity policies (https://www.nature.com/srep/journal-policies/editorial-policies#digital-image).

We have reviewed the figures provided with the manuscript and must ask that the following editorial requests are addressed during revision:

• We note that the images of the original blots appear to be still closely cropped. Are you able

to provide images showing full length membranes, with membrane edges visible, for this? Were the blots cut prior to hybridisation with antibodies? If original images of full-length blots cannot be provided, please include images of all blots as they are, with membrane edges visible, and for all replicates performed in the Supplementary Information file and include an explanation for the absence of images of adequate length where appropriate in the manuscript (i.e. methods and/or figure legends).
>Some of images (Fig 1A, Fig 1F, Fig 3D) did not include full length membranes, with membrane edges visible. I add the explanation in methods.

• We note that some of the figures are not accurately delineated. Delineation with dividing lines should be reserved for when non-contiguous lanes from different parts of the same gel, or from different gels, fields, or exposures are grouped together in the figures.
> All images were blotted from the same gel.
However, the exposure time for some images were not enough for visualizing lines.

• We note that some of the images display very high contrast. High contrast (overexposure) of gels and/or blots is discouraged. If it is unavoidable, please include multiple exposure images in the Supplementary Information file.
>Most images did not have multiple exposure images. What should I do?

• While we appreciate the inclusion of the original data in the manuscript, we note that it is very difficult to match some of these images to the figures (Fig 1A - caspase3, 23PRL, 16PRL and Fig 3D - Stat3, 23PRL) in the manuscript. Please ensure that the original images are clearly labelled so that they can be easily matched to the cropped versions in the manuscript figures. We also suggest denoting the regions of the original blots used in main figures using red boxes.
> We add red boxes in the regions of the original blots.

・The original image provided for Figure S1E-PRL does not seem to correspond to the cropped image in the manuscript figure. Please note that altering aspect ratios of blots for presentation purposes is not allowed. The cropped images shown in the main figures and their corresponding originals should also be versions taken at the same exposure level.

＞I changed the cropped image in the manuscript figure, as the same exposure level.

・Multiple originals have been provided for Figure 3D-PERK and p-PERK. Please clarify why. Where replicate (biological or technical) blots or multiple exposure images have been provided, please label these as such to avoid confusion.

> These are biological replicates. I add labels.

・We have noted that some of the original blots shown in the Supplement are over-exposed (e.g. originals of Figure 1A-p-PERK, PERK, GAPDH, Figure 1F - p-eIF2a, GAPDH, Figure 3D - GAPDH). High contrast (overexposure) of gels and/or blots is discouraged (please see attached file). If it is unavoidable, please include multiple exposure images (i.e. images of longer as well as shorter exposure) in the Supplementary Information file. If multiple exposure images cannot be provided, please include a justification and provide replicate blots if possible.

> In Figure 1A-p-PERK, PERK, GAPDH, I provide replicate blots.

In Figure 1F - p-eIF2a, GAPDH and Figure 3D - GAPDH, I provide multiple exposure images.
